# *Chlamydomonas* chloroplast genes tolerate compression of the genetic code to just 51 codons

**DOI:** 10.1073/pnas.2506263123

**Published:** 2026-01-06

**Authors:** Pawel M. Mordaka, Kitty Clouston, Jing Cui, Andre Holzer, Harry O. Jackson, Saul Purton, Alison G. Smith

**Affiliations:** ^a^Department of Plant Sciences, University of Cambridge, Cambridge CB2 3EA, United Kingdom; ^b^Department of Structural and Molecular Biology, University College London, London WC1E 6BT, United Kingdom; ^c^Holzer Scientific Consulting GmbH, Universität des Saarlandes, Saarbrücken 66123, Germany

**Keywords:** synthetic biology, chloroplast genome, genetic code, codon compression

## Abstract

Genome scale engineering has enabled codon compression of the universal genetic code to eliminate seven codons in *Escherichia coli*, but to allow more radical schemes for codon compression and reassignment to be tested at genome scale, while avoiding significant technical challenges, smaller, simpler genetic systems are needed. Here, we report a recoding scheme for the 205 kb *Chlamydomonas reinhardtii* chloroplast genome, in which two stop codons and one or more of the codons for arginine, glycine, isoleucine, leucine, and serine, all of which have two cognate transfer RNAs (tRNAs), are absent, compressing the genetic code to 51 codons. Several recoding strategies were tested on the essential *rpoA* gene, encoding a subunit of the chloroplast RNA polymerase. A defined compression scheme, which relied on swapping the target codons with the permitted frequent codons, could replace the native sequence without affecting expression of a reporter protein or strain fitness under standard laboratory conditions. The same strategy was successfully used for codon compression of *ycf1*, encoding a subunit of the chloroplast translocon, *psaA* and *psbA*, intron-containing highly expressed genes encoding reaction center subunits of both photosystems, and an 8.5 kb operon encoding essential and nonessential genes. Finally, we tested degeneracy of the 51-codon genetic code by exploring the combinatorial design for the large subunit of Rubisco, relying on restoration of photosynthesis in an *rbcL* mutant strain. More than 70 functional sequences with diverse codons were recovered. For all recoded genes, viable homoplasmic lines were obtained, showing the efficacy of our codon compression scheme.

## Significance

We demonstrate the use of the *Chlamydomonas* chloroplast genome as a synthetic biology platform to test radical compression of the universal genetic code. Genome engineering was used to generate strains in which 13 of the 64 possible triplet codes (codons) were eliminated from several essential and/or highly expressed genes, representing ~20% of the protein coding sequence in the genome. In all cases, viable and photosynthetically active strains were obtained, confirming the functional expression of the codon-compressed genes. These results pave the way for compression of the entire chloroplast genome to 51 codons, as well as the opportunity for more extreme modification or expansion of the genetic code.

The genetic code is universal and underpins all life on our planet. It provides the informational link between genes and their protein outputs, and therefore the instructions for building every organism. The code is simple and elegant—a set of 64 codons that code for 20 amino acids (AAs) and three stop commands—and, despite billions of years of evolution, has remained essentially unchanged among all extant life forms (1). The universality of the genetic code is perhaps surprising given that there are many ways in which AAs could be encoded using such a triplet code. Is the arrangement of the codon table the product of early optimization during the evolution of protein translation, or is it a “frozen accident,” with other arrangements of the table equally functional? The assignment of codons is clearly nonrandom, with related residues typically occupying contiguous codon boxes in the table, and a simple analysis of the code shows it to be highly robust to mutational or translational errors. It is debatable whether this reflects a selective force or is just a consequence of the codon table’s early evolution, and indeed theoretical analyses have identified some table variants more robust than the actual universal code (2, 3).

Testing completely different genetic codes is experimentally extremely challenging. However, improvements in custom DNA synthesis and DNA assembly in recent years have led to rapid advances in the field of synthetic genomics and allowed the design and synthesis of complete genomes for viruses, bacteria, and yeast (4, 5), and a region of the 450 Mb genome in the moss *Physcomitrium* (6). This has enabled moves toward modification of the genetic code. Successful examples of codon-compressed genomes have been reported, in which all instances of a particular codon have been modified to synonymous codons. In moss, a stop codon was removed from the synthetic region (6). In **Escherichia coli*,* first one or then two stop codons were removed (7, 8), and in *E. coli* Syn61, two sense and one stop codon were eliminated, enabling deletion of previously essential tRNAs and release factors (9). Most recently, *E. coli* Syn57 has been reported in which one stop and six sense codons were removed (9). In yeast, *Saccharomyces cerevisiae*, the Sc2.0 project has eliminated one stop codon from each of the 16 chromosomes, although not all in a single strain (10). Elimination of certain codons may then be used for reassigning the unused codons, and indeed this has been done to incorporate noncanonical amino acids for biosynthesis of unnatural polymers (10). A further benefit of such genome-wide codon reassignments is the opportunity to create robust genetic firewalls that result in resistance to viruses (10, 11) and improved biocontainment preventing horizontal gene transfer (12, 13).

Nonetheless, for the modified chromosomes and genomes assembled so far, even simple codon compression schemes were challenging to implement, requiring synthesis of Mb of DNA to change the several thousand codons in the genome. For **E. coli*,* initially eight different schemes were tested to introduce synonymous mutations in two sense codons to modify a 20 kb fragment encoding an operon rich in essential genes, genes expressed at a range of levels and membrane proteins (14) of which three schemes proved successful. When one of these schemes was applied to codon compression of the entire 3.9 Mb *E. coli* genome, modifying more than 18,000 codons, several sections needed to be redesigned to be functional and the recoded strain showed a significant fitness penalty, necessitating laboratory evolution to generate a robust strain that still grew 1.6 times slower than the WT (9). Recently, the same group tested more advanced codon compression schemes and were able to implement a 57-codon genome in *E. coli* (9), representing an impressive achievement where 101,605 codons were modified. To assemble genomes with more radical codon compression, allowing rapid iterative testing of reassignment and alternative genetic codes, a much smaller and simpler genetic platform would be desirable.

Chloroplast genomes are already highly reduced genetic systems, typically ranging between 120 and 210 kb depending on the species, and thus offer multiple advantages for testing codon compression and reassignment schemes. The 205 kb genome in the green alga *Chlamydomonas reinhardtii* encodes 70 protein genes (15) almost all of which have been identified and characterized through reverse genetic studies. High quality genome assemblies have been generated and transcripts have been mapped (15, 16). Only three genes have introns and these have been successfully replaced with intron-less versions, nor are there overlapping genes, which would otherwise complicate recoding designs. Moreover, unlike in vascular plants, there is no RNA editing in the *C. reinhardtii* chloroplast (15) reducing the risk of affecting the editing process by modification of the coding sequences. The chloroplast genome can be engineered by homologous recombination allowing precise manipulation and constructs for engineering can be rapidly assembled by using Golden-Gate technology (17). Most significantly, *C. reinhardtii* can dispense with photosynthesis if supplied with acetate as a carbon source (18), meaning that radical alterations of its genome that affect the photosynthesis genes are not necessarily lethal.

The highly reduced nature of the chloroplast genetic system is also reflected in the small number of tRNA genes encoded by the genome and used for protein synthesis. Chloroplast genomes rely on wobbling and superwobbling of codon–anticodon interactions to read the entire codon table, where some tRNAs can recognize up to four codons. In *C. reinhardtii*, there are 29 tRNA genes, compared to more than 280 tRNA genes in the nucleus (19). In the absence of import of nucleus-encoded tRNAs into the chloroplast, the minimum number of tRNAs that is necessary to translate all 61 sense codons could be as low as 25 (two tRNAs for leucine, isoleucine, serine, arginine, and methionine, and one for each of the remaining amino acids) (20). In this work, we explored the potential to significantly compress the genetic code of the *C. reinhardtii* chloroplast genome. We took advantage of the simplicity of the chloroplast genetic system and designed one of the most radical codon compression schemes tested to date. We successfully replaced several key genes with their codon-compressed variants and assessed the impact on chloroplast function.

## Results

### Establishing Design Principles for Codon Compression.

Analysis of the codon usage of the *C. reinhardtii* chloroplast genome (*SI Appendix*, Fig. S1) reveals a significant bias toward codons ending T or A, reflecting the high AT content (65.4%) of the plastome (15). Mapping the 29 tRNA genes (*Ct001-Ct029*) onto the codon table shows that the majority of amino acids are served by a single tRNA due to the wobble and superwobble in codon recognition (20, 21). Two tRNA genes are duplicated (*trnA-UGC*, *Ct013* and *Ct023*, and *trnI-GAU*, *Ct012* and *Ct024*) due to their location in the 22 kb inverted repeat (IR). There are also two copies of *trnE-UUC* (*Ct003* and *Ct0016*), which are identical across the length of the tRNA gene, but the upstream and downstream flanking sequences are different. This might be due to the involvement of *trnE* in tetrapyrrole biosynthesis as well as protein synthesis, creating an increased demand for this tRNA (16, 22, 23). Among the nonduplicated genes for tRNAs that read codons from the same codon box, there are two tRNAs for methionine, one for initiation (*Ct026*) and one for elongation (*Ct018*), and two isoacceptor tRNAs for glycine, *trnG-GCC* (*Ct011*) and *trnG-UCC* (*Ct021*). Previous experiments in *Nicotiana tabacum* showed that *trnG-UCC* is essential and can read all GGN glycine codons by superwobbling, whereas *trnG-GCC* can be deleted (24). It should also be noted that the amber TAG stop codon is used in only 4 of the 70 protein coding genes of *Chlamydomonas* and the opal TGA stop codon is not used at all (13).

The bias observed in the codon-tRNA landscape could be advantageous for compression of the genetic code as many of the rare codons could be replaced by frequently occurring synonymous codons without introducing a substantial number of codon changes in the genome. However, the reduced number of tRNAs in the chloroplast means that many of the rare codons are read by the same tRNA as other more frequently occurring codons within the same codon box. To allow reassignment of rare codons and avoid potential cross-talk, tRNAs could be modified to prevent wobbling. Alternatively, entire codon boxes could be modified, followed by deletion of the native tRNA gene.

**Fig. 1.**
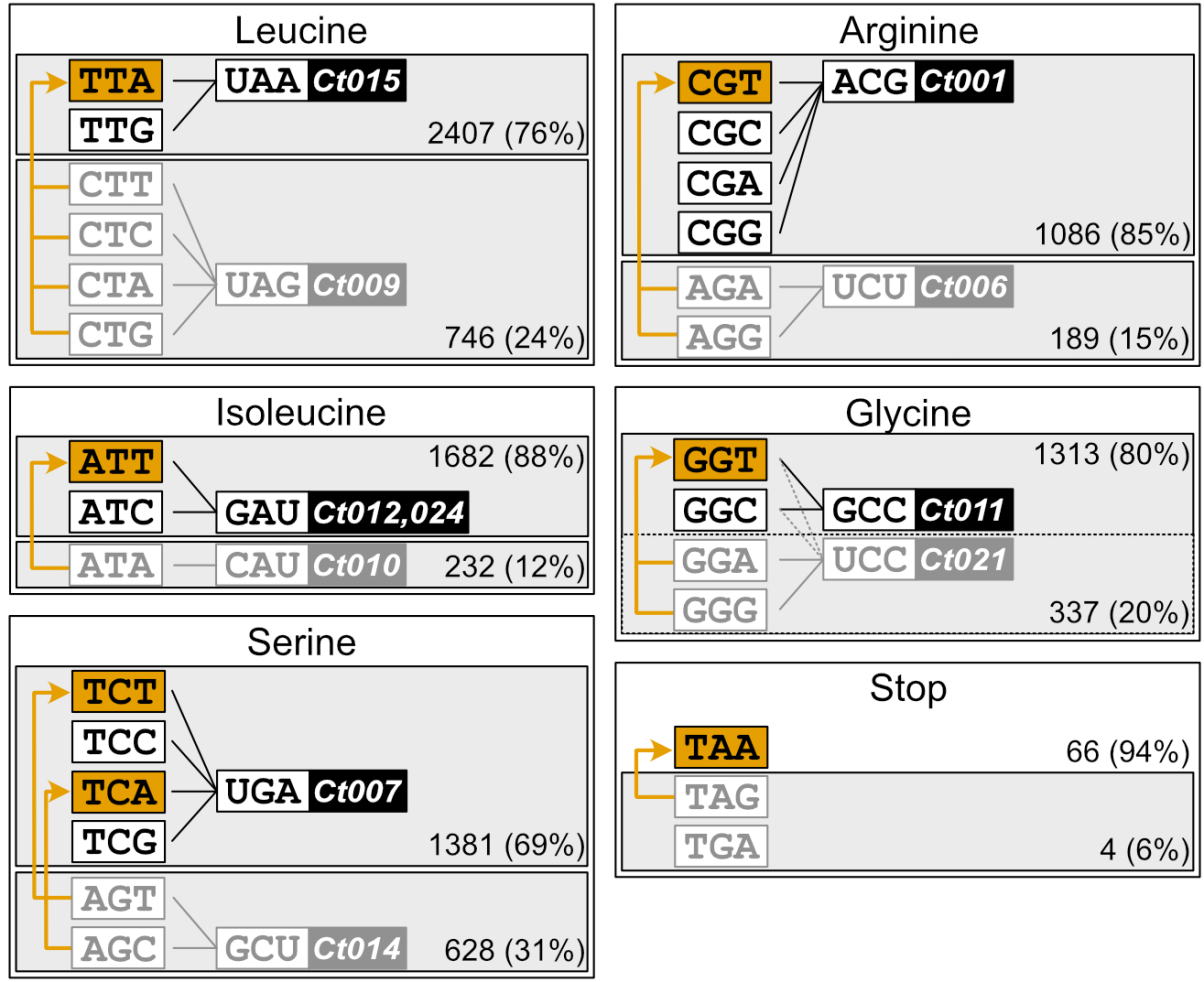
Proposed synonymous codon changes in the 51-codon defined compression scheme for the *C. reinhardtii* plastome. For each amino acid with two tRNAs with different anticodons (leucine, isoleucine, serine, arginine, and glycine), the target codons from the less frequently used codon box (gray) are replaced by the most frequent codon(s) from the other codon box (orange). In addition, four *amber* stop codons (TAG) are replaced by the *ochre* codons (TAA); the third stop codon (*opal*, TGA) is not present in the *C. reinhardtii* plastome. Numbers indicate the occurrence and frequency of all codons from each codon box.

We decided to take the latter approach for designing the genetic code compression scheme. For five amino acids (leucine, isoleucine, serine, arginine, and glycine), there are two tRNAs with differing frequency of use. For example, TTR codons that are read by tRNA-Leu(UAA) (Fig. 1, marked in black), occur 76% of the time, compared to 24% for CTN codons read by tRNA-Leu(UAG) (marked in gray). We hypothesized that for each of the five amino acids, the codons from the less frequently used codon box (target codons, marked in gray) could be changed to the most frequent codon(s) from the other box (marked in orange). After recoding all coding sequences in the chloroplast genome accordingly, together with changing the four amber (TAG) to ochre (TAA) stop codons, this would result in modification of 2,136 codons (7.5% of all total codons) and the genetic code would be compressed to just 51 codons.

### Testing the 51-Codon Scheme by Recoding Essential Genes.

Most of the chloroplast-encoded protein genes are short, with a median length of 190 codons, and use on average only 40 out of 63 codons, meaning that many do not contain rare codons and are therefore unsuitable proxies to infer the effects of the recoding schemes genome-wide (*SI Appendix*, Fig. S2*A*). Of the larger genes, that of *rpoA* (738 codons), encoding the alpha subunit of RNA polymerase, was chosen as the most suitable. It contains 57 unique codons, including at least one occurrence of 10 out of the 11 target sense codons for alteration in the compression scheme and has a frequency of these codons typical of the entire genome (7.5%, *SI Appendix*, Fig. S2*B*). The gene is expressed as a monocistronic mRNA (15), so its manipulation would not affect other genes located upstream and downstream. Previously, a 345 bp fragment of the *rpoA* gene containing the promoter and 5’UTR was successfully replaced with the promoter and 5’UTR of *psbD* gene via homologous recombination, and at the same time the protein was internally tagged with the FLAG epitope sequence for immunodetection (25), showing that the gene can be easily manipulated. In *C. reinhardtii*, the plastid-encoded RNA polymerase (PEP) is the only RNA polymerase present in the chloroplast, as the nuclear genome does not encode any chloroplast-targeted RNA polymerase (NEP) (26, 27), therefore all *rpo* subunits are essential for both photosynthetic and heterotrophic growth (25). As such, the *rpoA* coding sequence (CDS) is a good target to test for the effect of recoding, since suboptimal or no expression of the gene would lead to decreased chloroplast gene transcription or no growth.

**Fig. 2.**
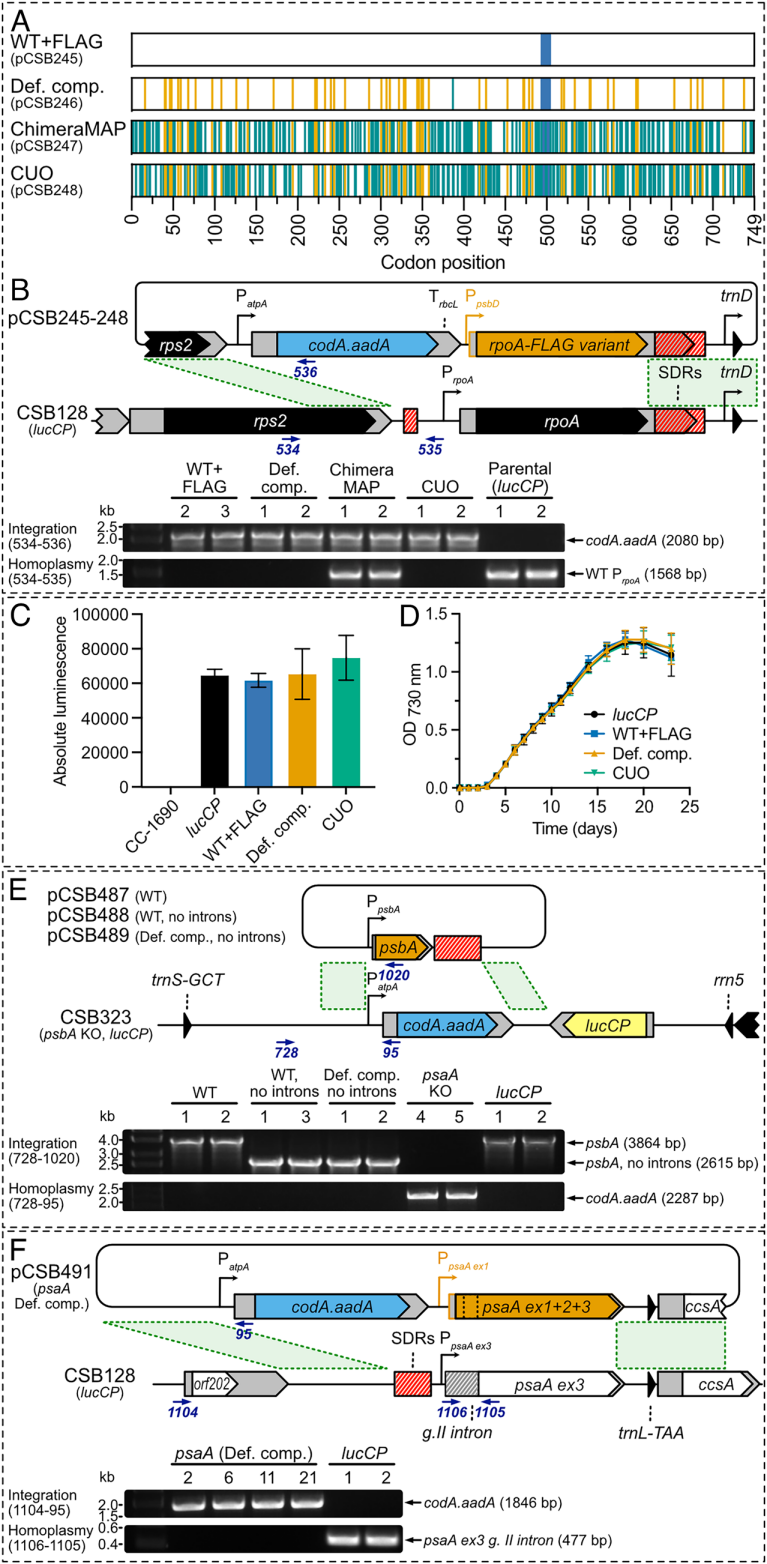
Codon compression of key genes in the *C. reinhardtii* plastome. (*A*) Schematic of *rpoA* sequences after codon compression by three different methods. Vertical orange lines represent synonymous mutations under the 51-codon defined compression scheme. Green lines represent other synonymous mutations (introduced to shorten the region of identity between the WT and defined compression variant or proposed by ChimeraMAP or CUO). The blue bar shows the position of the FLAG-tag. (*B*) *Top:* Schematic showing the replacement of the native *rpoA* coding sequence with the compressed variants encoded by plasmids pCSB246-248 via homologous recombination in the parental line CSB128 (which carries the *lucCP* cassette). Each plasmid replaced the 3.1 kb fragment between the left and right homology arms (green dashed lines) of the native *rpoA* gene and bordering Short Dispersed Repeats (SDRs, in red), with the *codA.aadA* cassette, and the recoded version of *rpoA* coding sequence, driven by the promoter/5’UTR of *psbD* gene. Also shown are the primers used for genotyping (blue arrows). *Bottom:* PCR of representative transformants using 534/536 primer pair to test for integration of the cassette or 534/535 primer pair to test for absence of the parental band, i.e., homoplasmy. (*C*) Luciferase activity in homoplasmic transformants for CSB245, CSB246, and CSB248 together with wild-type strain CC-1690 and the parental line, CSB128. Cells were grown in TAP medium to mid-logarithmic phase, harvested, normalized to OD_730nm_, and activity measured using the Steady-Glo Assay System (Promega, UK). (*D*) Phototrophic growth in cultures (25 mL) grown in HSM at light intensity of 80 μmol m^−2^ s^−1^. (*E*) Strategy to express *psbA* with the 51-codon defined compression, and without its four introns (CSB489). A native intron-containing sequence (CSB487) and a noncompressed control (CSB488) that just had the introns removed were also included. Plasmids pCSB487-489 were introduced into the *psbA* knock-out strain, CSB323, and transformants were selected by restoration of photosynthetic growth. (*F*) Strategy to express a 51-codon-compressed *psaA*. The three *trans*-spliced exons were combined, codon compressed, and cloned under the *psaA ex1* promoter/5’UTR together with the *codA.aadA* cassette in plasmid pCSB491. After transformation into CSB128, the plasmid replaced the 3.1 kb fragment spanning the *psaA ex3* promoter, group II intron and coding sequence, and bordering SDRs.

Three different codon-compressed *rpoA* coding sequences were designed and synthesized (Fig. 2*A* and Dataset S1). The “defined compression” scheme (cloned into plasmid pCSB246) replaced the 57 target codons, with the most frequent codons from the other codon boxes (marked in orange). To avoid a long 183 bp stretch of sequence homology between the native and the recoded gene that could act as a homology arm for recombination and potentially generate a chimeric WT-recoded gene, one other codon was modified (Lys387, rare AAG to frequent AAA, marked in green). The sequence included a FLAG-tag (blue box) to allow detection of the protein should homoplasmy not be reached (25). The other two sequence variants shown in Fig. 2*A* (pCSB247 and pCSB248) were fully designed in silico using two different codon optimization tools. ChimeraMAP (28) implemented in the CSO software (29) uses long substrings of codons that appear in the host coding sequences to retain genetic information embedded in the native sequence that conventional approaches cannot detect. Additionally, the Codon Usage Optimizer (CUO) software generates the synthetic sequence based on codons and codon pairs frequencies of a handpicked set of highly expressed genes in the chloroplast of *C. reinhardtii*. Both synthetic sequences resulted in changes in 176 codons when compared to the WT *rpoA* CDS, as some rare codons, both target (orange bars) and others (green bars), were preferentially replaced by more frequent codons. The sequences had a similar number of synonymous changes (346 and 339, 15.4% and 15.1% of all bases respectively), but the distribution of them was different, highlighting the contrasting approaches employed by the codon optimization tools.

These three codon-compressed *rpoA* sequences were used to generate a set of transformation plasmids (*SI Appendix*, Table S1). Each plasmid included the *codA.aadA* cassette (30) upstream of the target gene and homology arms to replace the native *rpoA* CDS with the compressed variant (Fig. 2*B*). To avoid undesired recombination between the 919 bp fragment of the *rpoA* promoter and 5’UTR (which is sufficiently long to act as a recombination homology arm, resulting in integration of the *codA.aadA* cassette without replacing the native *rpoA* CDS), the constructs used a 212 bp fragment containing the *psbD* promoter and 5’UTR. A control plasmid pCSB245 was identical apart from having the nonrecoded *rpoA* sequence. Plasmids were transformed into the chloroplast of *C. reinhardtii* strain CSB128 (*SI Appendix*, Table S2) containing the *lucCP* firefly luciferase cassette integrated in the *rrn5-psbA* integration sites in the inverted repeat (30). Spectinomycin-resistant transformants were obtained for all plasmids and 16 to 22 independent lines per plasmid were subcultured four times in increasing concentrations of antibiotic. Successful integration of the *codA.aadA* cassette was confirmed by colony PCR using a forward primer binding to the *rps2* gene located upstream of *rpoA* and a reverse primer binding to the *codA.aadA* cassette (*SI Appendix*, Table S3), and homoplasmy was tested by the absence of the WT *rpoA* PCR product. Two representative transformants for each construct are presented in Fig. 2*B*, with the remaining shown in *SI Appendix*, Fig. S3. The lines from the defined compression CSB246 constructs all successfully reached homoplasmy, while the frequency was 58% for the CUO CSB247 cell lines (*SI Appendix*, Table S4). Given the essential nature of the RpoA subunit, it can be concluded that these two designs allowed efficient translation of the protein. In contrast, no homoplasmic transformants were obtained for ChimeraMAP CSB248, suggesting that the design was not successful.

The expression of the *lucCP* cassette integrated into the chloroplast genome of the parental strain CSB128 provided a simple proxy to test whether recoding the *rpoA* sequence had any significant impact on the function of the protein in chloroplast transcription. CC-1690 (WT, parental strain of CSB128), CSB128, and homoplasmic lines of CSB245, CSB246, and CSB248 were grown in TAP medium, cells were harvested in the mid-logarithmic phase of growth and luciferase activity was tested (Fig. 2*C*). No statistically significant differences in luciferase activity were seen between the engineered strains. Moreover, photoautotrophic growth in minimal medium (HSM) under standard laboratory conditions (25 mL cultures in Nunc flasks at 25 ˚C and 80 µmol m^−2^ s^−1^) was essentially identical between the homoplasmic lines and the CSB128 parental strain (Fig. 2*D*). This confirmed that recoding of *rpoA* in CSB246 and CSB248, as well as *rpoA* promoter/5’UTR replacement and incorporation of a FLAG-tag, did not affect the function of the RNA polymerase, chloroplast gene expression or cell growth under these standard conditions.

One CSB246 line was retransformed with plasmid pCSB322 (*SI Appendix*, Fig. S4*A*) to restore the *rpoA* promoter and 5’UTR in front of the codon-compressed *rpoA* CDS, using the *aphA6* antibiotic cassette for selection. Transformants were selected on agar plates supplemented with kanamycin and then restreaked on medium supplemented with kanamycin and 5-fluorocytosine (to counterselect the *codA.aadA* cassette present in CSB246). Genotyping showed that cassette integration was homoplasmic in 15 out of 16 analyzed CSB322 lines (*SI Appendix*, Fig. S4*B* and Table S4). Complete recoding of *rpoA* was confirmed by next-generation amplicon sequencing (*SI Appendix*, Fig. S4*C*) using five overlapping primer pairs that do not distinguish between WT and recoded sequences and so were locus-independent. Together with the PCR-genotyping data, the results confirmed that the WT coding sequence was absent at the *rpoA* locus, as well as in other regions of the plastome or the nuclear genome. A growth assay showed that the codon-compressed *rpoA* CDS was also functional under the native promoter/5’UTR regulation (*SI Appendix*, Fig. S4*D*).

The efficacy of the defined compression scheme was further confirmed by recoding of the *ycf1* gene, encoding a second essential protein, the Tic214 subunit of the translocon of the inner chloroplast membrane (31). This has 1996 codons and a frequency of 7.1% target codons (*SI Appendix*, Fig. S2*B*). A total of 147 codons in the 6 kb *ycf1* gene were altered according to the 51-codon defined compression scheme with additional changes (*SI Appendix*, Fig. S5*A* and Dataset S1) and the synthesized DNA used to generate plasmid pCSB416 (*SI Appendix*, Fig. S5*B*). Transformation of CSB128 with this construct was used to replace the WT *ycf1* CDS. Of 18 transformants selected, 14 were shown to be homoplasmic (*SI Appendix*, Fig. S5*C* and Table S4), and sequencing confirmed replacement with the recoded gene (*SI Appendix*, Fig. S5*D*). This indicated that the recoded gene was functional. There was no observable impact of the codon compression on phototrophic growth as assessed by measuring growth in minimal medium as above (*SI Appendix*, Fig. S5*E*).

### Recoding of Highly Expressed Photosynthetic Genes Containing Introns.

We next investigated whether highly expressed genes could similarly be compressed, choosing those for the two reaction center proteins of photosystems I and II. *psbA* encodes the D1 protein of PSII, which is the most highly expressed and rapidly turned-over protein in the chloroplast (32). The gene contains four introns, but these are not essential for *psbA* expression since the WT CDS can be replaced by an intron-less version (33). The *psbA* gene is present in the 22 kb inverted repeat and so is in two copies. We generated an IR deletion mutant strain CSB358 (*SI Appendix*, Fig. S6*A*), using a similar strategy to that described for tobacco (34), which was still photosynthetic, and then a *psbA* knockout strain CSB323, by replacing the sole *psbA* gene with the *codA.aadA* cassette. As expected, the mutant was found to be nonphotosynthetic. To test recoded variants of *psbA*, the mutant was complemented by transforming the knockout line with plasmids pCSB487 encoding WT *psbA*, pCSB488 encoding WT *psbA* with no introns, and pCSB489 encoding recoded *psbA* with no introns (Fig. 2*E*). By plating the transformation on minimal medium, selection was by restoration of photosynthesis. Transformants were obtained for all three plasmids, with the presence of the *psbA* CDS variants confirmed by PCR genotyping and sequencing. Homoplasmy was obtained for multiple transformants (Fig. 2*E* and *SI Appendix*, Fig. S7*A* and Table S4). Growth assays in minimal medium under standard laboratory conditions showed no difference between the variants or the parental IR-deletion strain, CSB358 (*SI Appendix*, Fig. S7*B*).

PsaA, the A1 subunit of PSI, is also highly expressed, and uniquely is encoded by three exons distributed around the plastome that are *trans*-spliced, requiring an additional small chloroplast RNA gene, *tscA*, from a separate locus for splicing (34). We synthesized a DNA fragment spanning the entire CDS, combining the three exons, in which the 44 target codons (5.9%) were modified using the defined compression scheme. This was included in plasmid pCSB491, which was designed to replace the *psaA* exon 3 group II intron and exon 3 CDS with the recoded variant of the full-length CDS (Fig. 2*F*). In the engineered line, CSB491, exons 1 and 2 are present and transcribed, but since the exon 3 intron for assembling the entire processed transcript is missing, the only functional transcript for synthesis of the A1 protein would be the recoded intron-less *psaA*. Homoplasmic lines were obtained (*SI Appendix*, Fig. S8*A* and Table S4), and photoautotrophic growth under standard laboratory conditions was identical to the parental strain, CSB128 (*SI Appendix*, Fig. S8*B*), showing that the combined and recoded gene is functional.

### Recoding of the *trnM^e^-psbE-rps9-ycf4-ycf3-rps18-rps2* Operon.

**Fig. 3.**
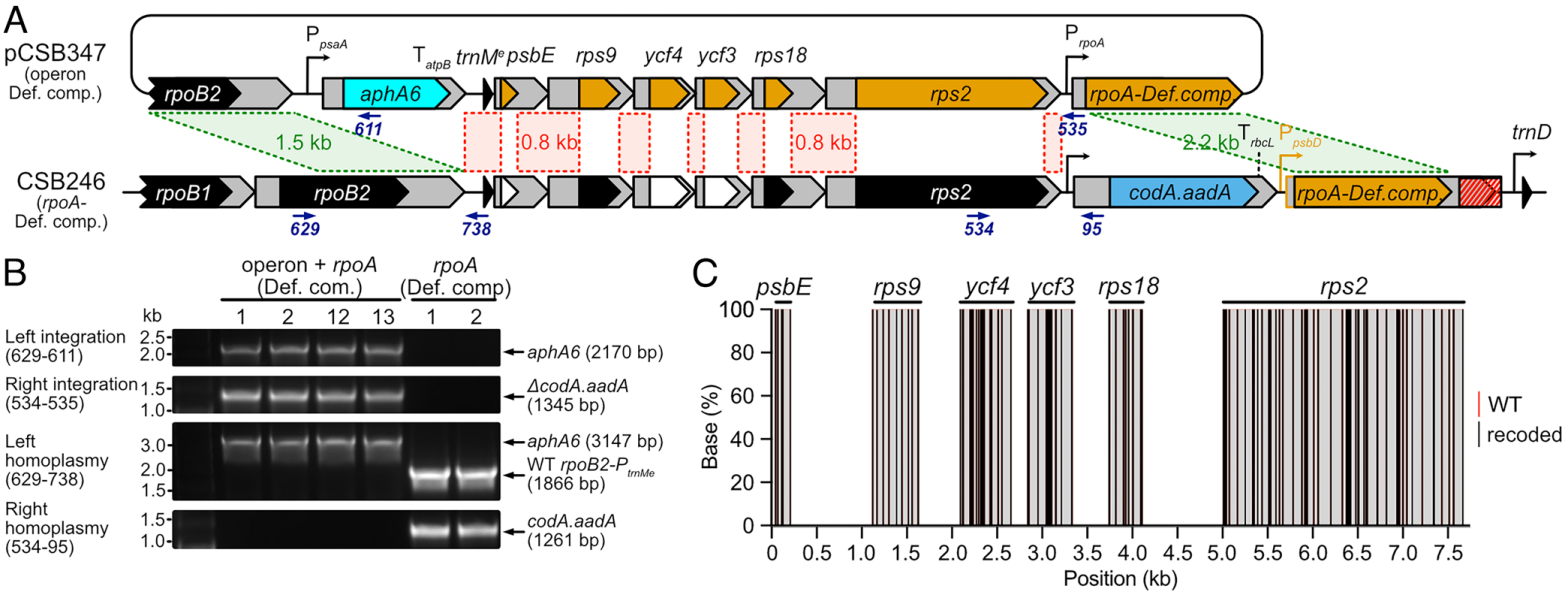
Codon compression of the *trnM^e^-psbE-rps9-ycf4-ycf3-rps18-rps2* operon. (*A*) Schematic showing the process of recoding the operon by transformation of the plasmid pCSB347 into strain CSB246-1 (with *rpoA* compressed by the defined compression scheme). The plasmid replaced the 11.6 kb fragment between the 1.5 kb left and 2.2 kb right homology arms (green dashed lines) with the *aphA6* cassette and the recoded versions of the coding sequences. At the same time, the *codA.aadA* cassette was deleted and the *rpoA* promoter/5’UTR was restored upstream of the *rpoA* CDS. Red dashed lines represent noncoding fragments of the operon identical in pCSB347 and CSB246. Primers used for genotyping are represented by blue arrows. (*B*) PCR analysis of representative transformants confirming integration of the entire *trnM^e^* operon and homoplasmy. (*C*) Next Generation Sequencing of the *trnM^e^* operon in line CSB347-1. The operon sequence was PCR-amplified as sixteen 450 bp overlapping fragments with primers annealing outside of the modified codons and sequenced by Illumina Paired-End sequencing (Azenta, UK). Data represent the frequency of the recoded (black) and WT bases (red) at target positions. Average frequency of recoded bases was 99.91%. Frequency of WT bases was consistent with sequencing error (0.03%). Sequencing coverage: minimum 2.7 k *x*, average 28.7 k *x*.

Having successfully recoded single coding sequences without generation of chimera, we sought to codon compress a larger fragment of the chloroplast genome. As a test case we selected the 8.5 kb fragment located upstream of the already recoded *rpoA* gene, encoding a cluster of genes transcribed together from the *trnM^e^* promoter (Fig. 3*A*). Besides the essential *trnM*^e^, this operon encodes three ribosomal subunit genes (*rps9*, *rps18,* and *rps2*) that are essential for plastid maintenance and three nonessential photosynthesis-related genes (*psbE*, *ycf4,* and *ycf3*). Coding sequences of these six genes were codon-compressed according to the defined 51-codon scheme, with alterations to 94 target codons (Dataset S1). To reduce regions of homology, up to ten additional codons were altered at one or both ends of the coding sequences (*SI Appendix*, Fig. S9*A*). The operon also contains the 5’ and 3’ UTRs, which are essential for the processing of the primary transcript and gene expression. Since mutations therein might result in altered expression of each gene, these sequences (up to 0.8 kb) were not modified so could result in generation of chimeric operons.

Plasmid pCSB347 encoding the recoded operon and the kanamycin resistance cassette was transformed into CSB246-1 (Fig. 3*A*). Homology arms in pCSB347 were designed in such a way that successful transformation would result in integration of the kanamycin cassette at the 5’ end of the operon and deletion of the *codA.aadA* cassette upstream of the already codon-compressed *rpoA*. Therefore, transformants were selected on kanamycin and 5-fluorocytosine and restreaked twice to reach homoplasmy. 40 independent transformants were analyzed and all showed correct integration of the kanamycin cassette, and 32 of them showed loss of the *codA.aadA* cassette (Fig. 3*B* and *SI Appendix*, Fig. S9*B* and Table S4). Coding sequences of four representative homoplasmic lines were amplified and sequenced to confirm successful replacement of the WT sequences with the recoded versions. Additionally, one line was further investigated by next generation amplicon sequencing as before, and the analysis showed that the operon was fully recoded (Fig. 3*C* and Dataset S2). There was no obvious difference in photosynthetic growth in minimal medium under standard conditions compared to CSB128 (*SI Appendix*, Fig. S9*C*).

### Further Characterization of the Recoded Strains.

Our focus in this work was to test whether a radical, 51-codon compression scheme could be used to recode essential and/or highly expressed genes in the chloroplast, as a prelude to recoding the entire plastome. In each of the examples described above, we were able to recover viable homoplasmic strains, with no apparent growth penalty under standard conditions (25 °C and 80 µmol m^−2^ s^−1^). Nonetheless, it is possible that the recoding might impact the ability of the chloroplast to withstand stress conditions. Accordingly, we conducted dilution spot tests for three independent transformants of each construct on agar plates and grew them under high light (500 µmol m^−2^ s^−1^ at 25 °C) and high light, high temperature (500 µmol m^−2^ s^−1^ at 35 °C) as well as standard conditions for comparison. *SI Appendix*, Fig. S10 shows the results after 7 d for the recoded *rpoA* (CSB246, CSB248, and CSB322) and *ycf1* (CSB416) strains alongside the parental CC-1690 and CSB128 (carrying the *lucCP* cassette) and strains made by the same method but with WT versions of the *rpoA* or *ycf1* gene (CSB245 and CSB317 respectively). In addition, strain CSB347 with a recoded *trnM^e^-rps2* operon, which is derived from CSB246 (*rpoA* recoded) is shown. Growth is observed to be essentially the same for all strains under each condition, with some slight reduction in growth under the most severe stress (500 µmol m^−2^ s^−1^ at 35 °C).

We carried out similar spot tests for the recoded *psbA* and *psaA* strains (*SI Appendix*, Fig. S11). No apparent differences were seen for the latter, but for the *psbA* lines, which were selected by restoration of photosynthetic growth of a Δ*psbA* mutant (strain CSB323) a reduction in growth under the high light or high light, high temperature conditions was seen for that complemented both by the recoded *psbA* (CSB489) and by the native WT gene including its introns (CSB487). To investigate this further, growth curves in liquid culture (4 mL in 6-well plates) were carried out for these strains under the three conditions (*SI Appendix*, Fig. S12*A*). The high light, high temperature condition resulted in essentially no growth for any of the strains, whereas for high light, the maximum cell density achieved for all strains was actually more than under standard conditions. Nonetheless, a decrease in growth rate during the exponential phase was observed for the recoded *psbA* strain (CSB489). This may be the result of a reduction in photosynthetic efficiency, measured as Fv/Fm (*SI Appendix*, Fig. S12*B*).

We were also interested to establish whether there were any unrelated genome rearrangements or gene duplications as a result of the transformations to introduce the recoded genes that would not be evidence from the next-generation amplicon sequencing. We therefore carried out whole genome sequencing using Oxford Nanopore Technology (ONT) and were able to construct de novo assemblies of the modified plastome for each of the strains (*SI Appendix*, Table S5). *SI Appendix*, Fig. S13 shows the reads obtained for strain CSB347 mapped to the reference parental genome (CSB128; *Top* panel) or to itself (*Bottom* panel). The expected modifications for the recoded *rpoA* and operon are visible as green bars in the top track when compared to the parental strain (feature 1), along with the FLAG-tag (feature 2) and antibiotic resistance gene (feature 4). These data show that despite the strain having undergone three rounds of transformation (to insert the *lucCP* cassette, to insert the recoded *rpoA*, and to insert the recoded operon) there are no other modifications in the plastome. Similar findings were obtained from sequencing the recoded *ycf1* (*SI Appendix*, Fig. S14) and *psbA* (*SI Appendix*, Fig. S15) genes. For strain CSB491, where the entire *psaA* coding sequence had been inserted into the *psaA ex 3* locus, although the recoded sequence is detected, some reads did not align contiguously to the reference sequences (feature 6), indicating the presence of a mixed population of two genome variants, the additional one resulting from recombination between the two copies of the *psaA ex1* promoters and 5’UTR (controlling expression of the native *psaA ex1* and the recoded intron-less *psaA ex1+2+3*), resulting in inversion of a 41.4 kb fragment of the plastome. However, this does not change the fact that the recoded *psaA* gene without introns is the only one that is functional in the plastome.

### Randomization of the 51-Codon Targets Using a Combinatorial Approach.

**Fig. 4.**
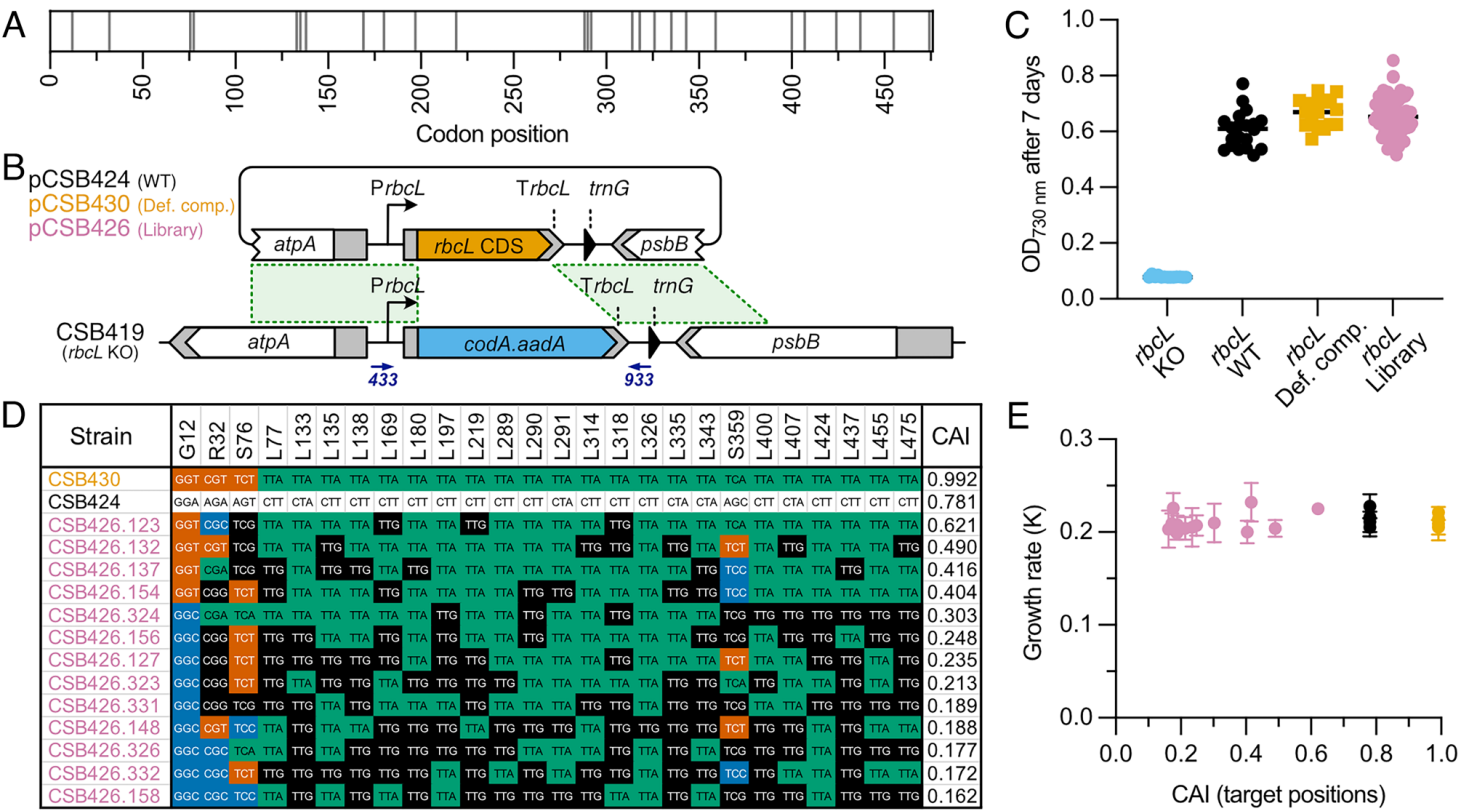
Codon compression of *rbcL* using the combinatorial approach. (*A*) Schematic of *rbcL* coding sequence containing 476 codons. Vertical gray lines represent positions of 26 target codons. (*B*) Schematic for recoding of *rbcL* coding sequence by complementation of the *rbcL* knockout strain CSB419 with plasmids pCSB424 (encoding the WT *rbcL* CDS), pCSB430 (defined compression scheme), or the pCSB426 plasmid library (combinatorially assembled *rbcL* variants). Primers used for genotyping and sequencing are represented by blue arrows. (*C*) Growth in minimal medium of the *rbcL* KO (CSB419, n = 13) and complemented strains (CSB424, n = 20; CSB430, n = 16; and CSB426, n = 73). Cells were grown in 96-well plates (culture volume 200 μL) in HSM at light intensity of 80 μmol m^−2^ s^−1^ and OD_730nm_ was measured after 7 d. Data points represent the average of three independent growth experiments. (*D*) Composition of the 26 target codons in 13 transformants selected from the CSB426 library compared to those in the WT *rbcL* (CSB424) and *rbcL* compressed using the defined compression scheme (CSB430). Sequences are sorted based on the decreasing value of the CAI calculated for the 26 target positions. Randomized codons are colored by the third base of the codon: T—red, C—blue, A—green, G—black. (*E*) Growth rate of the CSB424 (n = 4), CSB430 (n = 4), and CSB426 (n = 13) transformants in minimal medium plotted against the CAI of the target positions. Cultures (25 mL) were grown in flasks for 3 wk. OD_730nm_ of cultures was measured every 24 h and the growth rate (K) was calculated after fitting data points to the Gompertz growth function (GraphPad Prism, v10). Error bars represent SD for three independent cultures.

The recoding carried out so far changed the target codons to defined codons (indicated in orange in Fig. 1). However, even after compression to 51 codons, the genetic code remains degenerate, allowing translation from a single tRNA for each of serine and arginine from four different codons (TCN and CGN, respectively) and leucine, isoleucine, and glycine from two different codons (TTR, ATY, and GGY, respectively). In each case these have quite different codon usage frequencies, up to 90-fold (e.g., arginine CGT vs. CGG; *SI Appendix*, Fig. S1). We therefore sought to explore the effect of using each of the codon options in the compression scheme. We hypothesized that codon usage may be especially important for expression of genes for highly abundant proteins, so chose *rbcL*, encoding the large subunit of RuBisCO, as a test subject. This gene contains 26 target codons for four of the amino acids (22 leucine, 2 serine, 1 arginine, and 1 glycine; Fig. 4*A*). Replacing these with all possible options from the 51-codon compression scheme (i.e., both orange and black codons in Fig. 1) would result in 5.4 × 10^8^ unique coding sequence variants. This would be virtually impossible to screen as independent lines, so instead, we decided to use a complementation assay to test a library of randomly generated codon-compressed variants of *rbcL* for their ability to rescue an *rbcL* mutant.

First, an *rbcL* knockout line CSB419 (*rbcL* KO) was generated by replacing the *rbcL* CDS with *codA.aadA* (*SI Appendix*, Fig. S17). As expected, the mutant was found to be nonphotosynthetic, and in this case light-sensitive. A library of complementation plasmids (pCSB426) was then generated by incorporation of synthetic degenerate oligonucleotides in which the *rbcL* sequence was randomized at the positions of the 26 target codons to introduce synonymous mutations (*SI Appendix*, Fig. S18). Bulk sequencing of the plasmid library showed detectable degeneracy at the expected base positions (*SI Appendix*, Fig. S19), with one exception, L326, where 99% were one codon (TTA). Control plasmids encoding the WT *rbcL* CDS (pCSB424) and the *rbcL* recoded using the defined compression scheme (pCSB430) were also assembled (Fig. 4*B*). These were transformed into CSB419 and lines encoding a functional *rbcL* were selected by plating on minimal medium that supports only photosynthetic growth. Transformation efficiency was different between the control plasmids (40 to 80 colonies per µg DNA) and the pCSB426-lib (4 to 5 colonies per µg DNA), presumably reflecting the fact that not all library variants encoded a correctly assembled CDS. To reach homoplasmy, colonies obtained for each construct were then restreaked on minimal medium supplemented with 5-fluorocytosine, to counterselect the CSB419 chloroplast DNA containing the *codA.aadA* marker. After genotyping by PCR, the *rbcL* CDS was sequenced in 83 independent CSB426 lines. Of these, we eliminated from further analysis ten lines that were photosynthetic but had additional silent or missense substitutions at nontarget positions. The remaining 73 *rbcL* sequences had unique combinations of synonymous mutations only at the target positions (*SI Appendix*, Fig. S20). Photosynthetic growth, measured as OD_730nm_ after 7 d, was determined in a high-throughput experiment in 96-well plates in minimal medium (Fig. 4*C*). As expected, unlike the parental strain CSB419 (*rbcL* KO), the CSB424, CSB430, and CSB426 transformants were able to grow and showed similar end-point optical densities.

To characterize the complemented strains in more detail, the proportion of frequent (ending with A or T) to rare codons (ending with G or C) was examined (*SI Appendix*, Fig. S20) and a subset of 13 library transformants was taken forward (Fig. 4*D*). They spanned the range of frequent:rare codon ratios from 21:5 to 8:18 and the set also included those showing the highest and the lowest OD_730nm_ values from the 96-well plate experiment and also sequences containing all possible permitted codons (four for serine and arginine and two for glycine and leucine) present at each randomized position. To illustrate the synonymous codon usage bias of selected sequences, the Codon Adaptation Index (CAI) (35) for the target positions was calculated (Fig. 4*D*). The selected lines were grown in 25 mL cultures for 3 wk (*SI Appendix*, Fig. S21*A*) and the growth rates (K) for each line calculated and plotted against the CAI value (Fig. 4*E*). No significant differences between the growth rates of the codon-compressed lines and the controls were observed under the standard laboratory conditions. We then further tested the *rbcL* defined compression variant (CSB430) under high light, and high light plus high temperature (*SI Appendix*, Fig. S21*B*), compared to the parental strain (CC-1690) and the *rbcL* mutant complemented with the WT *rbcL* gene (CSB424). Again, compression of the genetic code for *rbcL* did not limit the photosynthetic growth even under stress conditions. No large-scale genomic alterations were detected in CSB430 using ONT sequencing (*SI Appendix*, Fig. S21*C* and Table S5).

## Discussion

We have taken a step toward generation of an entire *Chlamydomonas* chloroplast genome with a radically compressed genetic code. We took advantage of the simplicity of the highly reduced genetic system of the plastome and designed a compression scheme to reduce the genetic code to just 51 codons. We tested the scheme by recoding and successfully replacing several native genes fulfilling different chloroplast functions, including essential genes required for the transcription–translation machinery (*rpoA, rps2, rps9, rps18*), an essential gene encoding a subunit of the membrane translocon complex (*ycf1*), and highly expressed genes encoding subunits of the photosynthetic complexes (*psaA, psbA, psbE,* and *rbcL*). In total, we compressed more than 18 kb of coding sequence (21% of the plastome) and modified 377 target codons (17.6% of all target codons in the plastome). Despite the significant compression of multiple key genes, none of the engineered strains suffered from loss of cell fitness, as evidenced by the ease of reaching homoplasmy after transformation and WT-like growth under photoautotrophic conditions.

When designing the 51-codon compression scheme, we focused on targeting the codons for amino acids having two codon boxes and synonymously mutating the codons from the less frequently used codon box (2,136 target codons, 7.5%). Using similar principles, even more radical codon compression schemes to just 46 codons are possible, (e.g., by eliminating arginine CGN, isoleucine ATY, and serine TCN codons) at a cost of larger number of codon swaps (5,236 codons, 18.4%). A codon compression scheme to just 20 sense codons has been previously proposed and used with moderate success for recoding of a small subset of essential yeast genes (22 out of 25 CDS remained functional after recoding) (36, 37). Whether this would be possible for the entire genome, where gene families or genes with common domains would have repeats of identical nucleotide sequences, remains unknown.

In codon compression schemes tested in *E. coli*, the target codons were replaced by codons with the closest match as calculated by either the CAI, the tRNA adaptation index or translation efficiency (14), but using these metrics did not predict optimal recoding schemes. In *Chlamydomonas*, the effect of using rare vs. frequent codons on expression of the highly turned-over PsbA subunit has been investigated (38). Incorporation of certain rare codons (e.g., arginine CGG or AGG) impaired protein production, whereas the presence of others did not (e.g., glycine GGG or alanine GCG). In some cases (a serine rare codon cluster of 3xTCC), impairment of PsbA synthesis and ribosome pausing was observed, but only at high light intensities (1,500 μmol m^−2^ s^−1^), suggesting that translation speed is not a limiting factor under standard growth conditions (50 to 100 μmol m^−2^ s^−1^). These effects are difficult to infer from the codon usage table and are likely to be context specific. Our defined codon compression scheme replaced target codons with the most frequent permitted codon(s), in case there were any negative effects of rare codons. The marginal slowing of the growth rate in exponential phase of the *psbA* recoded strain (CSB489) seen in *SI Appendix*, Fig. S12*A* under high light might be due to other reasons, such as alterations in mRNA secondary structure or its stability. Recently, high-resolution ribosome profiling of the *psbA* transcript revealed that translation of the PsbA protein slowed down between codons 135 and 150. The authors proposed this was due to a ribosome open-ratchet formation or binding of a regulatory factor to the transcript (39). However, this region of the *psbA* coding sequence does not contain any target codons for the 51-codon compression scheme, so was not modified in our construct.

We explored further the tolerance for frequency of use of codons using a combinatorial approach to identify which permitted codons are acceptable, this time in the highly expressed photosynthetic gene, *rbcL*. After complementation of the *rbcL* knockout with the library of coding sequences, 73 unique functional sequences were identified (Fig. 4*C*). They contained all possible codons at all positions and showed different numbers of frequent and rare codons. However, none of the compressed sequences caused a detectable limitation of RbcL synthesis as shown by the growth assay. Also, mutating just 26 codons had only a minor effect on the CAI for the entire coding sequence (change from 0.77 to 0.70 in the variant encoding only rare codons). Because of the nature of the complementation experiment, only functional recoded sequences could be identified. In a situation when an attempt to recode a particular gene is unsuccessful, the native target codons could be included in the combinatorial design to identify positions that need troubleshooting.

During the design of the *E. coli* Syn61 strain, additional identification and refactoring of 79 gene overlaps were necessary to allow their independent recoding (9). Moreover, recoding of two hypothetical ORFs (*yceQ* and *yaaY*) located within 5’UTRs of adjacent essential genes had negative effects on the regulation of these genes and needed further redesign. In the end, the resulting strain had a 1.6-fold increase in the doubling time, indicating that recoding had reduced fitness overall. The recently completed *E. coli* Syn57, where one stop codon and six sense codons have been eliminated, also required significant trouble-shooting to remove all WT sequences and laboratory evolution to increase fitness, with the final strain still exhibiting severely reduced growth compared to the nonrecoded strain (9). A different project to construct an *E. coli* strain with similar codon compression was able to recode 39% of the genome, but was unable to reach completion (40).

Further issues during recoding of *E. coli* and yeast genes were generation of new sequences with promoter activities and intragenic transcription initiation caused by the synonymous mutations, resulting in generation of antisense transcripts (37, 40). These transcripts may affect gene regulation and therefore active elimination of de novo promoters during genome design is necessary. Regulation of the gene expression in the chloroplast occurs not at the transcription initiation stage, but rather at the transcript processing stage, when different RNA binding proteins coordinate stabilization, maturation, and degradation of RNA (41, 42), possibly making the chloroplast gene regulation less sensitive to spurious promoters. Also, attempts to generate chloroplast synthetic promoters by randomization of an existing strong promoter sequence resulted in only 10% capable of driving detectable expression of the reporter gene (43). Generation of synthetic promoter libraries for other AT-rich genomes showed that such organisms may have evolved more stringent recognition of promoter sequences to avoid initiation of transcription at spurious sites (44), and this would reduce the chance of generating promoters de novo during codon compression of the entire genome. The effect of such extensive recoding on the chloroplast transcriptome will require further investigation in the future.

Beyond studies of the genetic code itself, there is considerable interest in generating and introducing a completely synthetic genome, and the small size of the plastome makes it attractive for this purpose. Previously, it was shown that chloroplast genomes can be assembled and propagated in heterologous hosts (45, 46). However, a successful replacement of the native genome with the synthetic version resulted in chimeric sequences likely due to the polyploid nature of the chloroplast genome and the high level of homologous recombination (45). Efficient recombination can occur between fragments of identical sequence as short as 200 bp (30). We found that recoding genes according to the defined 51-codon compression scheme makes the coding sequence sufficiently different to the native coding sequences and allows complete replacement of the genome fragments. In our initial experiments, we introduced additional mutations to reduce the lengths of identical sequences between the codon-compressed and the native fragments (e.g., Fig. 2*A* and *SI Appendix*, Fig. S5*A*). Analysis of a few randomly selected homoplasmic transformants did not reveal any chimeric sequences resulting from recombination. Subsequently, we found that even larger recoded fragments of ~9 kb were efficiently incorporated, despite the presence of long unmodified noncoding fragments (Fig. 3*A*). Here, double selection was used by selecting for the integration of the positive marker (*aphA6*) at the left junction and deletion of a negative marker (*codA*) at the right junction. This method could be used iteratively to replace the entire genome with recoded sequences, as in the assembly of the *E. coli* Syn61 and Syn57 genomes (8, 9, 15).

In conclusion, the simplicity of the chloroplast genome as a minimal genetic system offers a versatile synthetic biology platform for exploring compression, rearrangement, and expansion of the genetic code. The results presented here highlight the potential of the genome as a valuable system for radical genetic code compression and reassignment efforts and justify the efforts to synthesize the entire recoded genome. Once the chloroplast genome is fully recoded, the five less frequently used tRNA genes (*trnL-UAG Ct009, trnI-CAU Ct010, trnS-GCU Ct014, trnR-UCU Ct006, and trnG-UCC Ct021*) would become nonessential and could be deleted. In the next step, the genetic code could be modified in different ways by reassigning the unused codons to any one of the 19 alternative canonical amino acids addressing the question of whether the code is a product of early optimization or the result of chance. In the *Chlamydomonas* chloroplast there is only one amino acyl tRNA synthetase for each of the two tRNAs serving the five amino acids, but the anticodon sequences are different for the two tRNAs in each case, so there must be some tolerance of anticodon changes. Ultimately, the unused codons could also be reassigned to encode noncanonical amino acids for synthesis of new-to-nature proteins with novel properties, using orthogonal tRNA and amino acyl tRNA synthetases as reported in ref. 10.

## Materials and Methods

### Cultivation of *Chlamydomonas* Strains.

*C. reinhardtii* strains (*SI Appendix*, Table S2) were grown mixotrophically or heterotrophically in TAP medium (47) or photoautotrophically in minimal media, HSM (48), supplemented with trace elements (49), on plates with agar (2% w/v; Formedium Ltd, UK) or in liquid medium, either at 25 °C under constant illumination (80 μmol m^−2^ s^−1^) or in darkness (photosynthetic mutant strains only). Cultures set up in cell culture flasks (Nunc EasYFlask 25 cm^2^, filter closure, Thermo Scientific, UK) were grown in shaking incubators at 120 rpm (Infors Multitron, Infors UK Ltd), while cultures in 96-well microplates (volume 200 μL) were grown in stationary incubators. Optical density of liquid cultures was measured using a UV-VIS spectrophotometer (Thermo Scientific, UK) or CLARIOstar Plus plate reader (BMG Labtech, UK). Cell density was measured in C-Chip hemocytometer slides (Cambridge Bioscience, UK) using the Rebel hybrid microscope (Discover Echo, USA). Cultures were supplemented with spectinomycin (200 to 300 μg mL^−1^; Melford, UK), kanamycin (200 mg mL^−1^; Melford, UK), or 5-fluorocytosine (5 mg mL^−1^; Thermo Scientific, UK) as detailed in the text.

### Plasmid Design and Assembly.

Plasmids (*SI Appendix*, Table S1) were generated using modular cloning methods. Recoded coding sequences were designed by manual replacement of the target codons for the defined compression scheme or using codon optimization tools, ChimeraMAP (29) and CUO (Algal Biology and Biotechnology group, UCL; https://github.com/khai-/CUO, accessed on 05-08-2020), by setting the frequency of the target codons in the codon usage tables to 0. DNA fragments were synthesized as gene fragments (Twist Biosciences, USA) or cloned ValueGENE plasmids (Azenta, UK). Constructs for transforming the chloroplast genome were assembled using the Start-Stop Assembly method (50) and included flanking regions for homologous recombination at the appropriate loci. Reactions mixtures were transformed into chemically competent *E. coli* NEB5α cells (NEB, UK) followed by selection on LB agar plates with respective antibiotics (carbenicillin, 100 μg mL^−1^; tetracycline, 10 μg mL^−1^). Plasmids were extracted and purified using Monarch® Miniprep Kit (NEB, UK) for small scale cultures, or GenElute^TM^ HP Midiprep Kit (Sigma-Aldrich, UK) for medium scale cultures. Plasmid concentrations were determined using a NanoDrop spectrophotometer (Thermo Scientific, UK). All plasmids were verified by enzymatic digestion with appropriate restriction enzymes and sequence identity was confirmed by Sanger or Plasmid-EZ sequencing (Azenta, UK).

### Chloroplast Transformation of *Chlamydomonas*.

Transformation followed the protocol of ref. 51 using bombardment with a Biolistics PDS-1000/HE device (Bio-Rad Laboratories, UK), using 1,350 psi rupture discs (Bio-Rad Laboratories). Gold DNAdel carrier particles (Seashell Technology) were coated with the plasmid DNA at a concentration of 5 μg of DNA per 1 mg of gold particles, following manufacturer instructions. For each bombardment 2.5 μg of DNA was used. Transformants were selected on TAP agar plates supplemented with spectinomycin (200 μg mL^−1^; Melford, UK) or kanamycin (200 μg mL^−1^; Melford, UK), or on HSM agar plates (photosynthesis restoration). To reach homoplasmy, sixteen colonies per construct were streaked onto fresh selection plates, incubated for 7 d and then restreaked up to 3 times. Genotypes of transformed strains were confirmed by using the Phire Plant Direct PCR Kit (Thermo Scientific, UK) according to the manufacturer’s protocol. Transgene integration was verified using primers (*SI Appendix*, Table S3) annealing to the transgene cassette and to the genome flanking the integration site. Homoplasmy was verified using primers that specifically amplify the wild-type gene at the integration locus, such that no product was formed once all chloroplast genome copies contain the transgene.

### Luciferase Assay.

Luciferase assays were performed using Steady-Glo Luciferase Assay System (Promega, UK). Samples of cultures in logarithmic phase of growth (1 mL) were centrifuged at 1,000 × g for 10 min and pellets were resuspended in phosphate-buffered saline (PBS) to OD_730nm_ of 0.5. Samples (50 μL) were mixed with the lysis buffer (50 μL; 2 × Cell Culture Lysis Reagent Promega, UK, diluted in PBS) and incubated at room temperature for 10 min. Cell extracts (25 μL) were mixed with an equal volume of Steady-Glo® Reagent. Luminescence was measured at 580 ± 80 nm using CLARIOstar™ plate reader after 5 min incubation.

### NGS Amplicon Sequencing.

Total DNA was extracted from cell pellets using the SDS method described by ref. 52. Fragments of the chloroplast genome designated for NGS sequencing (450 to 500 bp) were amplified using the Platinum™ II Taq Hot-Start DNA Polymerase (ThermoFisher Scientific, UK) using primers with or without partial Illumina® adapter sequences (*SI Appendix*, Table S3). PCR products were column purified using illustra™ GFX™ PCR DNA purification kit (VWR, UK). DNA concentration was determined using Qubit dsDNA HS Assay Kit (ThermoFisher Scientific, UK). Samples were normalized to 20 ng μL^−1^, pooled, and sequenced using Amplicon-EZ service (Azenta, UK). Raw reads were trimmed to quality score 20, mapped to the reference sequence using Unipro UGENE 52.0 BWA-MEM mapping tool and nucleotide variants were counted (53).

### Long-Read Sequencing Using ONT.

Total DNA was extracted from cell pellets using the method described by refs. 27 or 54 with or without additional treatment with Short Read Eliminator XS (Pac-Bio), as detailed in *SI Appendix*, Table S5. Libraries were prepared with the Ligation Sequencing Kit (SQK-LSK114, Oxford Nanopore Technologies) and run on Flongle Flow Cells (FLO- FLG114). Base calling was performed using Dorado v7.8.3 HAC (minQ 9). Reads were mapped to the reference genomes using Unipro UGENE BWA-MEM (53) and alignments were visualized using IGV Desktop Application v2.19.5 (55). De novo assemblies were generated using the open-source pipeline, PlastomePipelineForONT (http://github.com/Sgt-Spaghetti/PlastomePipelineForONT, accessed on 29 August 2025). Within the pipeline, raw reads were filtered, retaining the reads with a GC content of 0 to 42%, a read length greater than 10 kb, and a mean quality score above 30. Assemblies were performed with the Flye assembler.

## Supplementary Material

Appendix 01 (PDF)

Dataset S01 (XLSX)

Dataset S02 (XLSX)

## Data Availability

Sequence data generated in this study are available as follows. Amplicon and Sanger Sequencing at https://github.com/pmmordaka/Plant_Metabolism_UCAM (DOI: 10.5281/zenodo.15053965) (56). ONT data and assemblies are at both European Nucleotide Archive (ENA) Project PRJEB96787 (https://www.ebi.ac.uk/ena/browser/view/PRJEB96787) (57) and at Zenodo (DOI: 10.5281/zenodo.17350679) (58). All other data are included in the manuscript and/or supporting information.
