# Supplementary material for: Chlamydomonas chloroplast genes tolerate compression of the genetic code to just 51 codons
Source: Proc Natl Acad Sci U S A. 2026 Jan 6;123(2):e2506263123. doi: 10.1073/pnas.2506263123 (PMC12799115; doi:10.1073/pnas.2506263123)
Supplement: Supplementary file 1 — Appendix 01 (PDF) [file pnas.2506263123.sapp.pdf]

Supporting Information for

Chlamydomonas chloroplast genes tolerate compression of the genetic code to just 51 codons

Pawel M. Mordaka, Kitty Clouston, Jing Cui, Andre Holzer, Harry O. Jackson, Saul Purton and Alison G. Smith

Corresponding author: Alison G Smith
Email: as25@cam.ac.uk

This PDF file includes:

Figures S1 to S21
Tables S1 to S5
SI References

Other supporting materials for this manuscript include the following:

Datasets S1 to S2
Data repository (Amplicon and Sanger Sequencing):
https://github.com/pmmordaka/Plant_Metabolism_UCAM
(DOI 10.5281/zenodo.15053965)
Data repository (ONT Sequencing):
ENA Project: PRJEB96787
Zenodo (DOI 10.5281/zenodo.17350679)

Supporting Figures

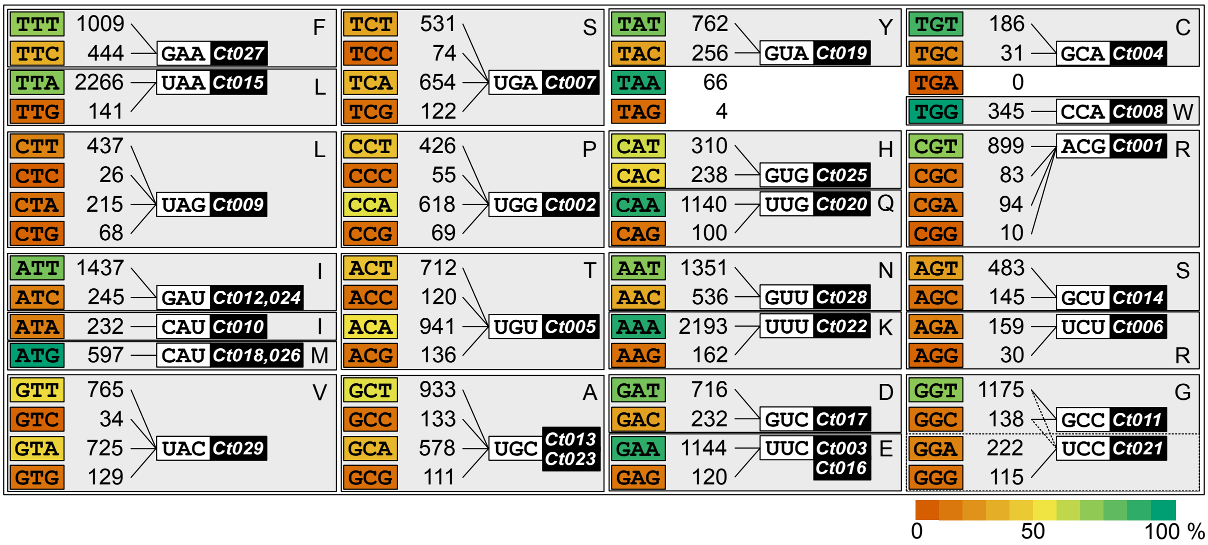

Fig. S1. Codon table and tRNAs of *C. reinhardtii* chloroplast genome. The absolute number of codons was calculated based on 70 unique open reading frames present in the chloroplast genome assembly (CPv4) for strain CC-503 (1). tRNA genes and anticodons were predicted by tRNAscan-SE 2.0 (2) and cross-referenced with the PlantRNA database (3). tRNA gene identifiers (*Ct001-029*) were taken from Cpv4 (1). Codon-anticodon interactions were predicted based on wobbling and superwobbling rules established experimentally for the plastome of *Nicotiana tabacum* (4) and bioinformatics analysis for *C. reinhardtii* (5). The heat-map represents the percentage of each codon among all codons encoding the same amino acid.

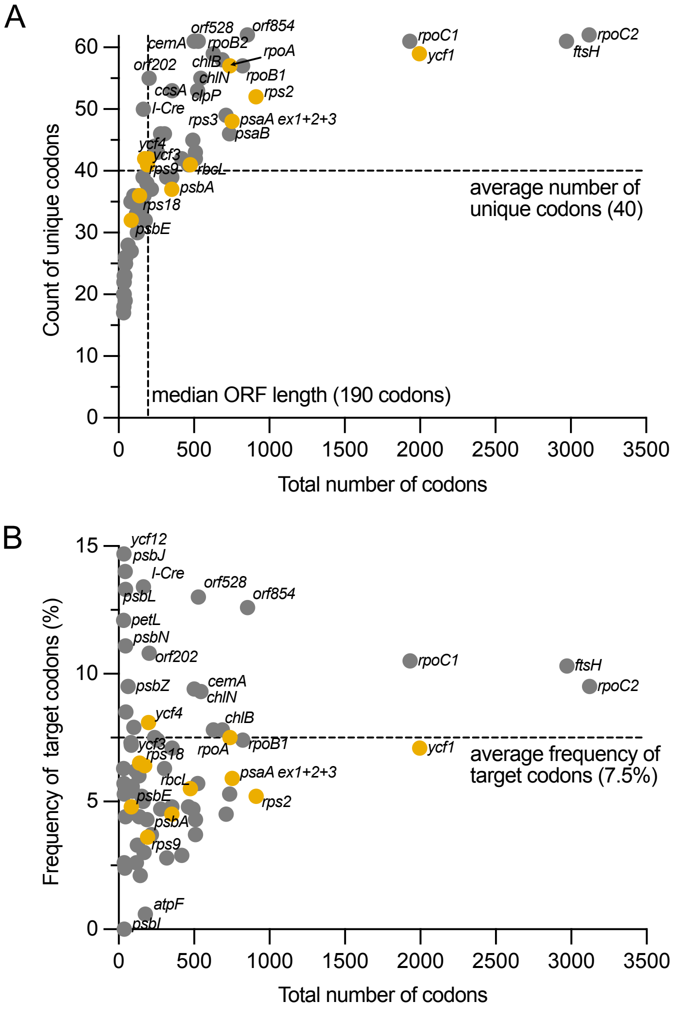

Fig. S2. Count of unique codons (**A**) and frequency of 51-codon compression scheme target codons (**B**) against the total number of codons in protein coding sequences in the *C. reinhardtii* chloroplast genome. Genes successfully recoded in this study are marked in orange.

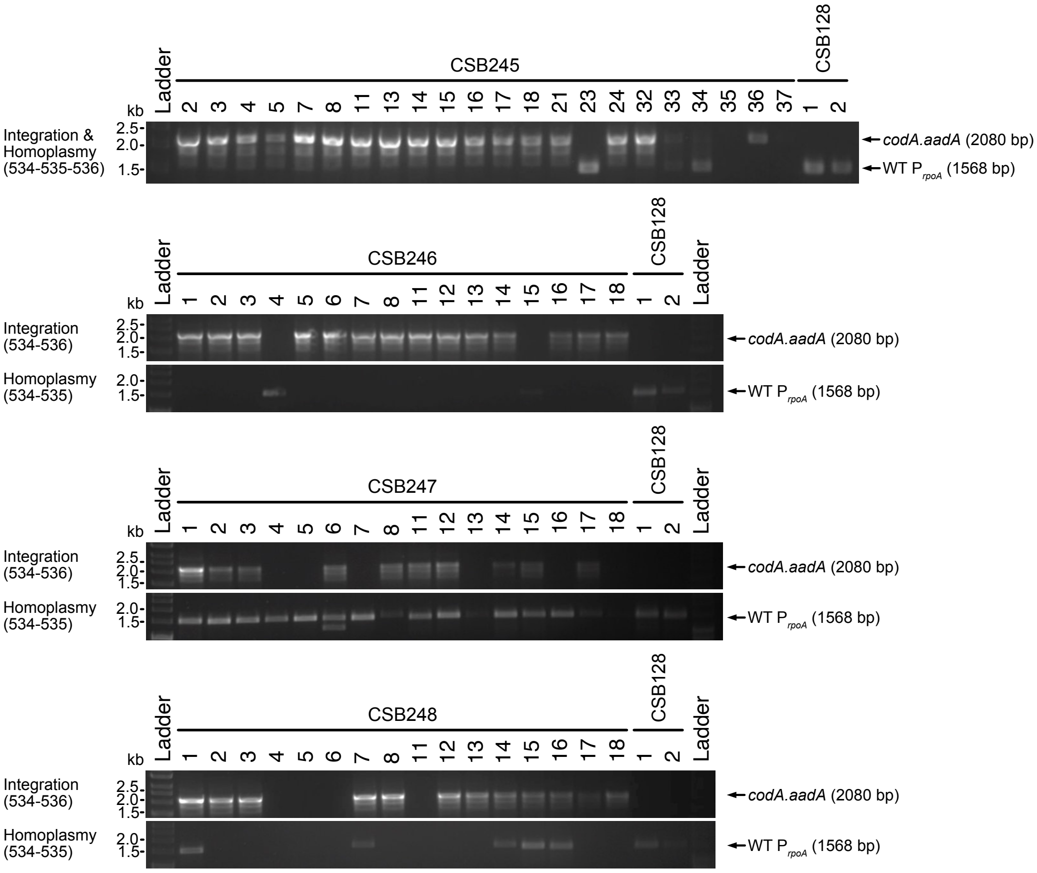

Fig. S3. Genotyping of *rpoA* recoding transformants. Integration of the *codA.aadA* cassette and homoplasmy was tested by PCR for lines transformed with plasmids pCSB245, pCSB246, pCSB247 and pCSB248. Primers used for amplification are listed on the left and sizes of the expected products on right. Parental strain CSB128 was used as a control. Ladder - HyperLadder^{TM} 1kb (Bioline).

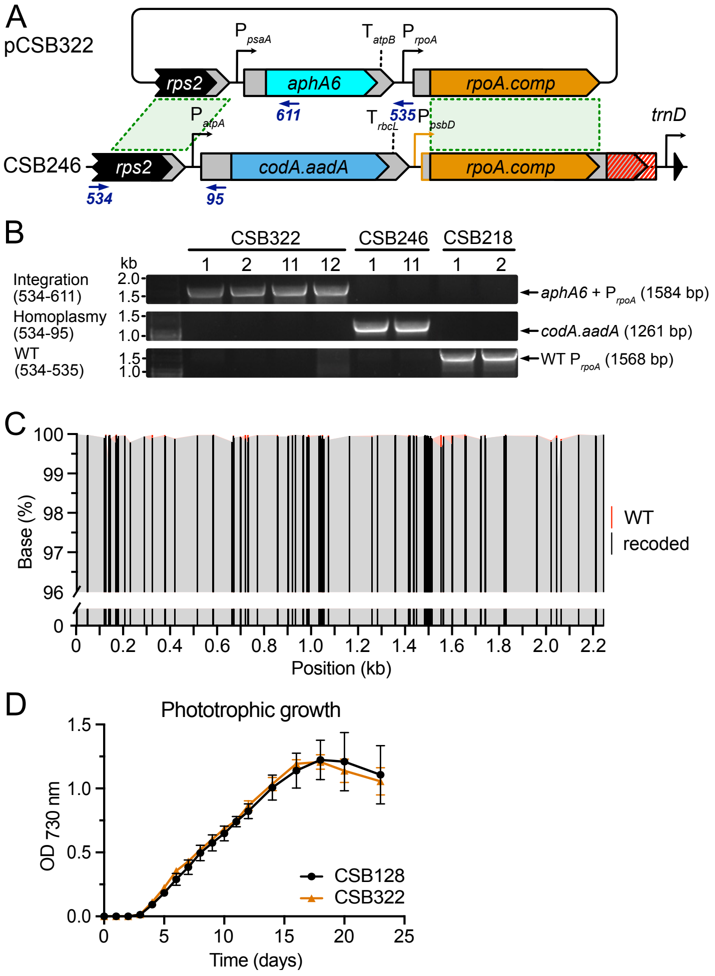

Fig. S4. Restoration of *rpoA* promoter and 5'UTR in CSB246. (A) Schematic showing the restoration of the *rpoA* promoter and 5'UTR upstream of the *rpoA* CDS recoded using the 51 defined compression scheme. The *rpoA* codon-compressed strain CSB246 was transformed with plasmid pCSB322 and transformants were selected on kanamycin and 5-FC. Primers used for genotyping are represented by blue arrows. **(B)** Genotyping of the representative transformant lines. Primers oligoCSB534 and oligoCSB611 were used for confirmation of the plasmid integration resulting in a product of 1584 bp. Primer pairs oligoCSB534-oligoCSB95 and oligoCSB534-oligoCSB535 were used for detection of the CSB246 and WT plastome DNA (products of 1261 bp and 1568 bp respectively). **(C)** Confirmation of *rpoA* recoding by Amplicon NGS. The entire coding sequence of *rpoA* was amplified as five overlapping 497 bp PCR products, pooled and pair-end sequenced using Illumina sequencing platform (Amplicon-EZ, Azenta). Bars represent positions of synonymous substitutions. Frequency of bases: black - recoded, red - WT. **(D)** Phototrophic growth of homoplasmic CSB322 lines in cultures (20 mL) grown in HSM under white light at 80 µmol·m^{-2}·s^{-1} compared to the parental CSB128 strain. Note this experiment was done at the same time as that for the CSB245 strain, so the control data are the same as in Fig. 2D.

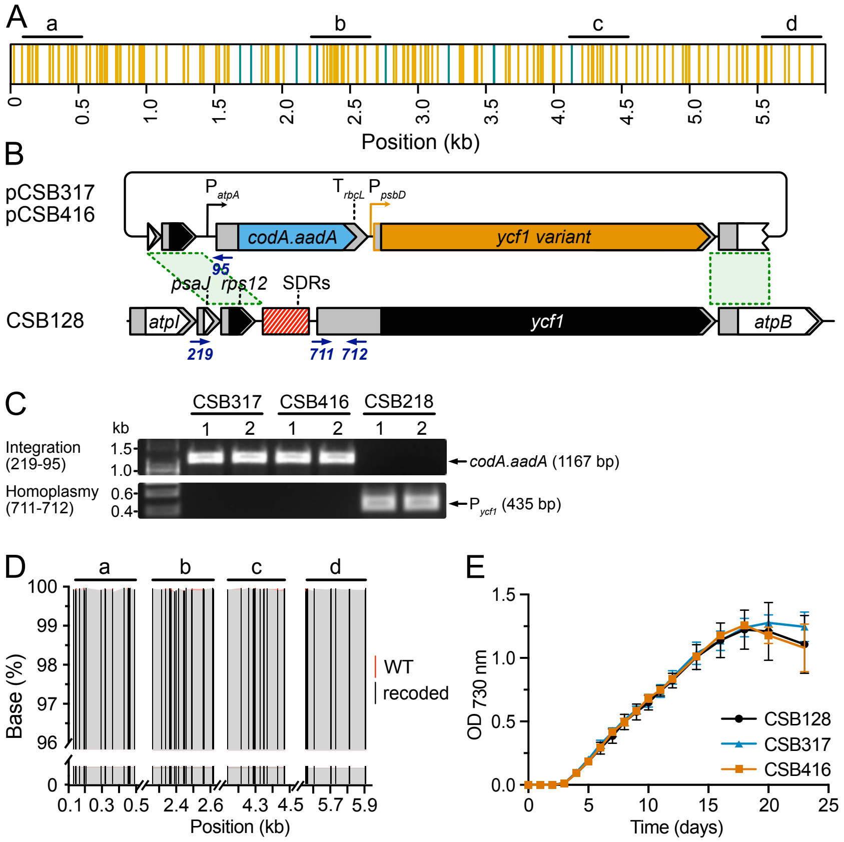

Fig. S5. Codon compression of the *ycf1* coding sequence. (A) Schematic showing the positions of target codons in the *ycf1* coding sequence. Vertical lines represent synonymous mutations in the coding sequence (orange - target codons, green - other codons). (**B)** Plastome recombination with plasmid pCSB416 used for recoding of the *ycf1* gene coding sequence. The plasmid replaced the 8148 bp fragment of the plastome located between the left and right homology arms (green dashed lines) containing Short Dispersed Repeats (SDRs, in red), promoter, 5'UTR and coding sequence of *ycf1* gene with the *codA.aadA* cassette, promoter and 5'UTR of *psbD* gene, and the recoded version of *ycf1* coding sequence. Plasmid pCSB317 was used as control, where the SDRs and *ycf1* promoter/5'UTR were replaced with the *codA.aadA* cassette and the promoter and 5'UTR of *psbD* gene. **(C)** Genotyping of the transformant lines. Primers oligoCSB219 and oligoCSB95 were used for confirmation of the plasmid integration resulting in a product of 1167 bp. Primers oligoCSB711 and oligoCSB712 were used for detection of the WT DNA (product of 435 bp). **(D)** Confirmation of *ycf1* recoding by Amplicon Next Generation Sequencing. Four fragments of the *ycf1* coding sequence were amplified as 450 bp PCR products, pooled and pair-end sequenced using Illumina sequencing platform (Amplicon-EZ, Azenta). Bars represent silent substitutions. Frequency of bases: black - recoded, red - WT. **(E)** Phototrophic growth of the homoplasmic transformant lines. CSB128 and transformants CSB317 and CSB416 were grown in HSM at light intensity of 80 µmol·m^{-2}·s^{-1} compared to the parental CSB128 strain. Note this experiment was done at the same time as that for the CSB245 strain, so the control data are the same as in Fig. 2D.

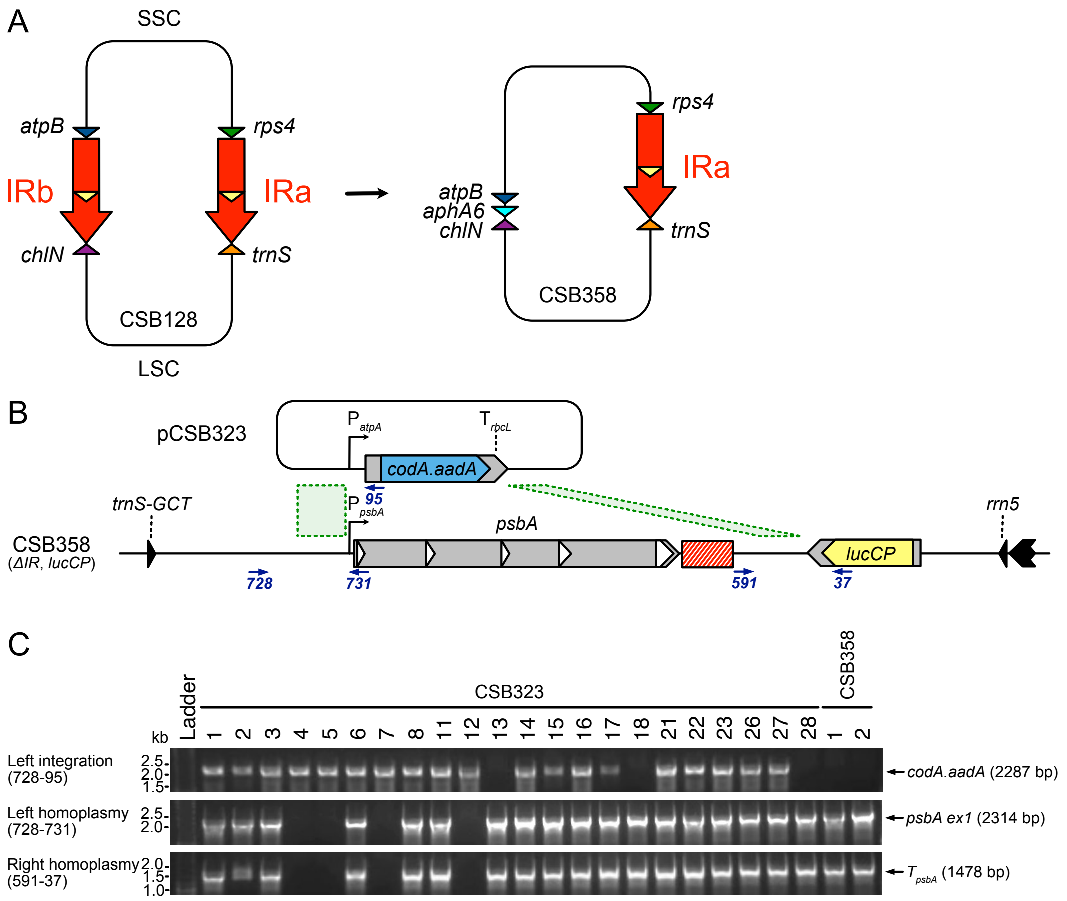

Fig. S6. Generation of the *psbA* knockout strain. (A) Schematic showing the plastome of strain CSB358. In CSB128 *lucCP* strain, the inverted repeat b (IRb) flanked by *chlN* and *atpB* genes has been replaced by the kanamycin resistance cassette *aphA6*. Size of the reduced plastome is 186,968 bp. **(B)** Schematic showing the process of deletion of the *psbA* gene by transformation of the plasmid pCSB323 into the strain CSB358. The plasmid replaced the 7.8 kb fragment between the left and right homology arms (green dashed lines) with the *codA.aadA* cassette. Primers used for genotyping are represented by blue arrows. **(C)** PCR analysis of transformants confirming homoplasmic deletion of *psbA*. Primers oligoCSB728-oligoCSB95 were used for confirmation of the plasmid integration resulting in a product of 2287 bp. Primer pairs oligoCSB728-oligoCSB731 and oligoCSB591-oligoCSB37 were used for detection of the WT DNA on left and right junctions (products of 2314 bp and 1478 bp, respectively).

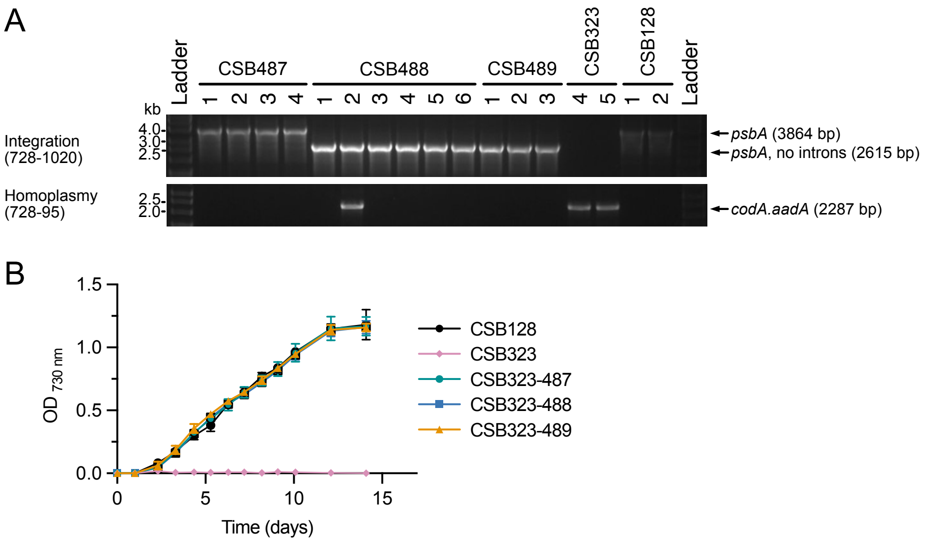

Fig. S7. Codon compression of *psbA*. (A) PCR analysis of transformants confirming reinsertion of the modified *psbA* in CSB323. Primers oligoCSB728 and oligoCSB1020 were used for detection of the *psbA* coding sequence resulting in products of 3864 bp (with introns) and 2615 bp (without introns). Primers oligoCSB728 and oligoCSB95 were used for homoplasmy screening by detection of the *codA.aadA* cassette (product of 2287 bp). **(B)** Phototrophic growth of the homoplasmic complemented lines and the parental lines. Cultures (n=3) were grown in HSM at light intensity of 80 μmol·m^{-2}·s^{-1}.

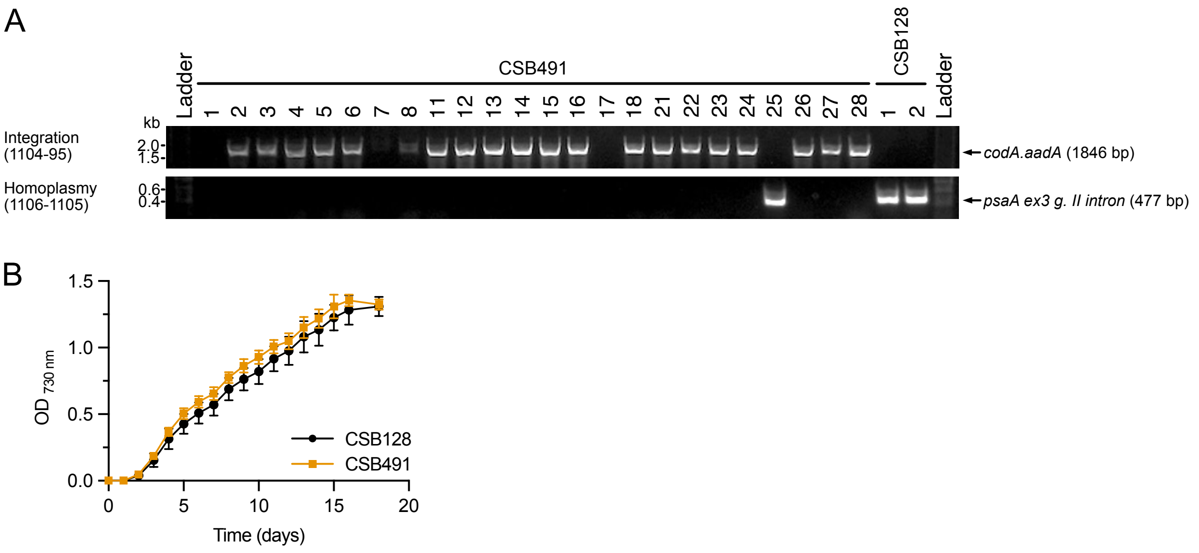

Fig. S8. Codon compression of *psaA*. (A) PCR analysis of transformants confirming recoding of *psaA* in CSB128. Primers oligoCSB1104 and oligoCSB95 were used for integration of the *codA.aadA* cassette resulting in a product of 1846 bp. Primers oligoCSB1106 and oligoCSB1105 were used for homoplasmy screening by detection of the *psaA ex. 3* group **II** intron (product of 477 bp). **(B)** Phototrophic growth of the homoplasmic complemented lines and the parental lines. Cultures (n=8) were grown in HSM at light intensity of 80 µmol·m^{-2}·s^{-1}.

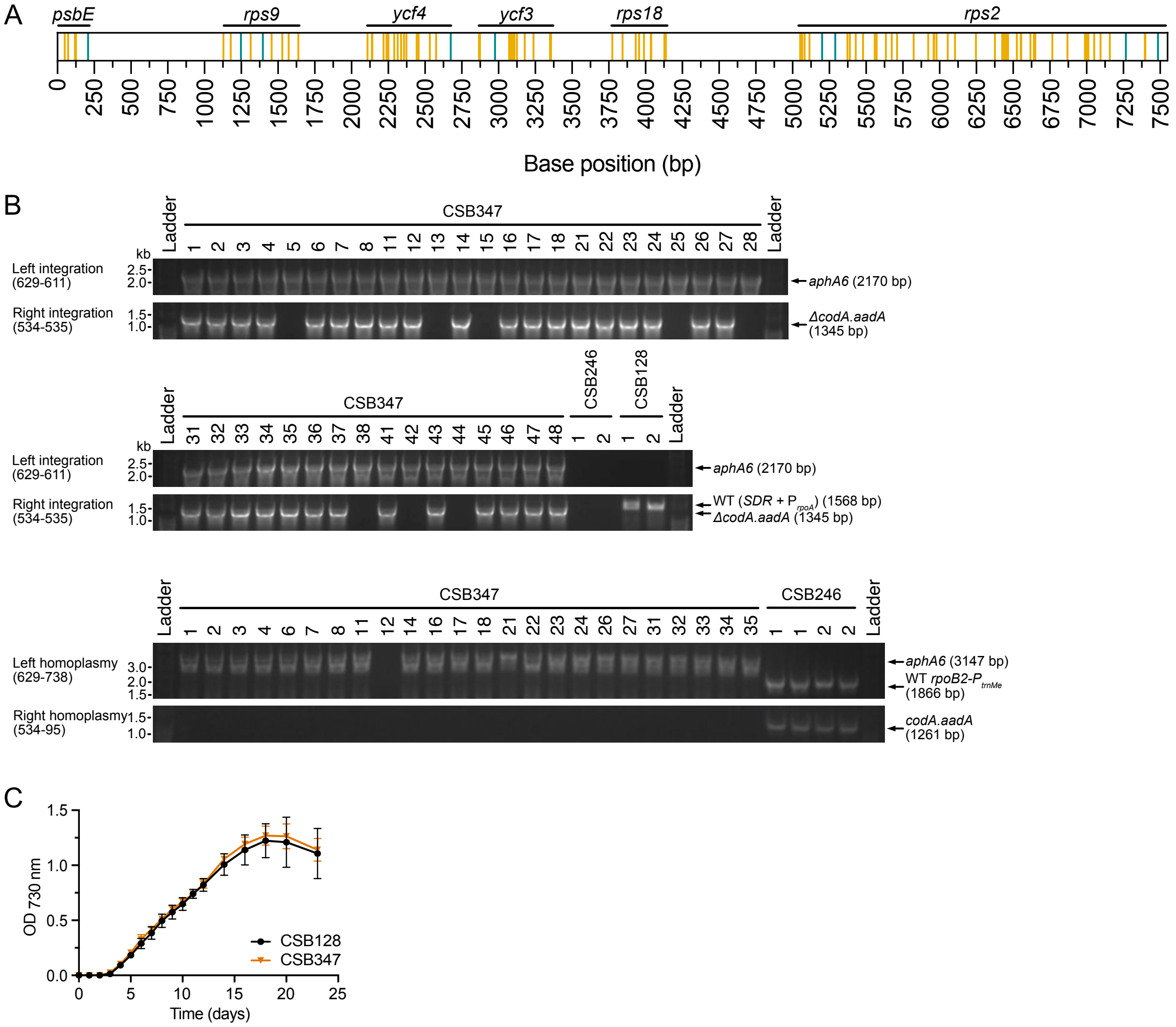

Fig. S9. Codon compression of the *trnM*^e^*-psbE-rps9-ycf4-ycf3-rps18-rps2* operon (A) Schematic showing the positions of target codons in the *trnM*^e^ operon. Coding sequences of individual genes are marked above. Vertical lines represent synonymous mutations in the coding sequences (orange - target codons, green - other codons). **(B)** PCR analysis of transformants confirming integration of the entire *trnM*^e^ operon (oligoCSB629-oligoCSB611 for the left integration junction and oligoCSB534-oligoCSB535 for the right integration junction) and homoplasmy screen (primers oligoCSB629-oligoCSB738 for the left junction homoplasmy and oligoCSB534-oligoCSB95 for the right junction homoplasmy). **(C)** Phototrophic growth of the representative homoplasmic lines and the parental line CSB128. Cultures (n=4) were grown in HSM at light intensity of 80 μmol·m^{-2}·s^{-1} compared to the parental CSB128 strain. Note this experiment was done at the same time as that for the CSB245 strain, so the control data are the same as in Fig. 2D.

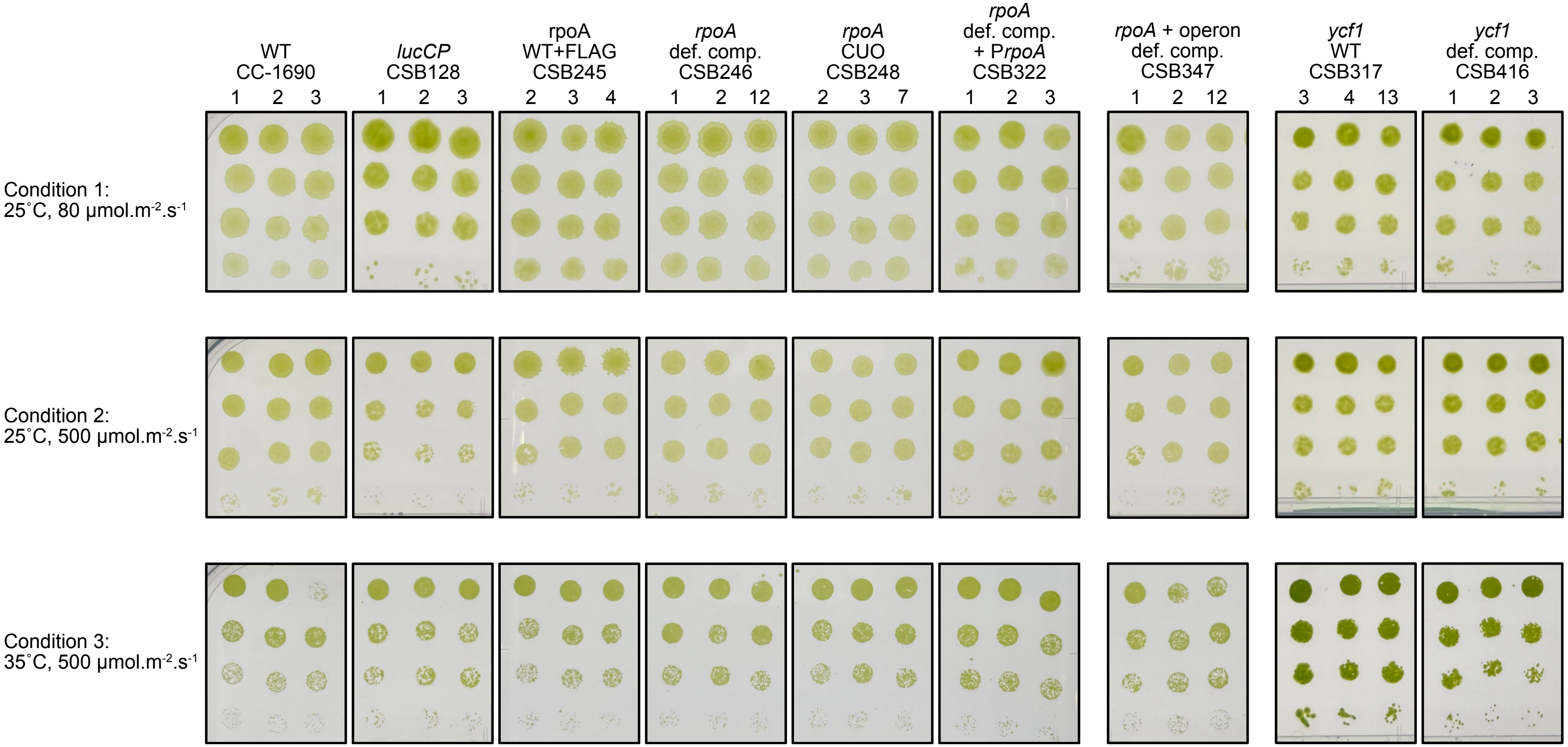

Fig. S10. Phototrophic growth of *rpoA*, *ycf1* and *trnMe-rps2* operon codon compressed strains at high light intensity and at high temperature. Cultures in the mid-logarithmic phase of growth (OD 0.5) were spotted on HSM plates and incubated under three growth conditions: 25˚C and 80 µmol $m^{-2}.s^{-1}$ (control), 25˚C, 500 µmol.$m^{-2}.s^{-1}$ (high light) and 35˚C, 500 µmol.$m^{-2}.s^{-1}$ (high light and high temperature). Dilution factors: 1, 5, 10, 100. Photographs were taken after 7 days of incubation.

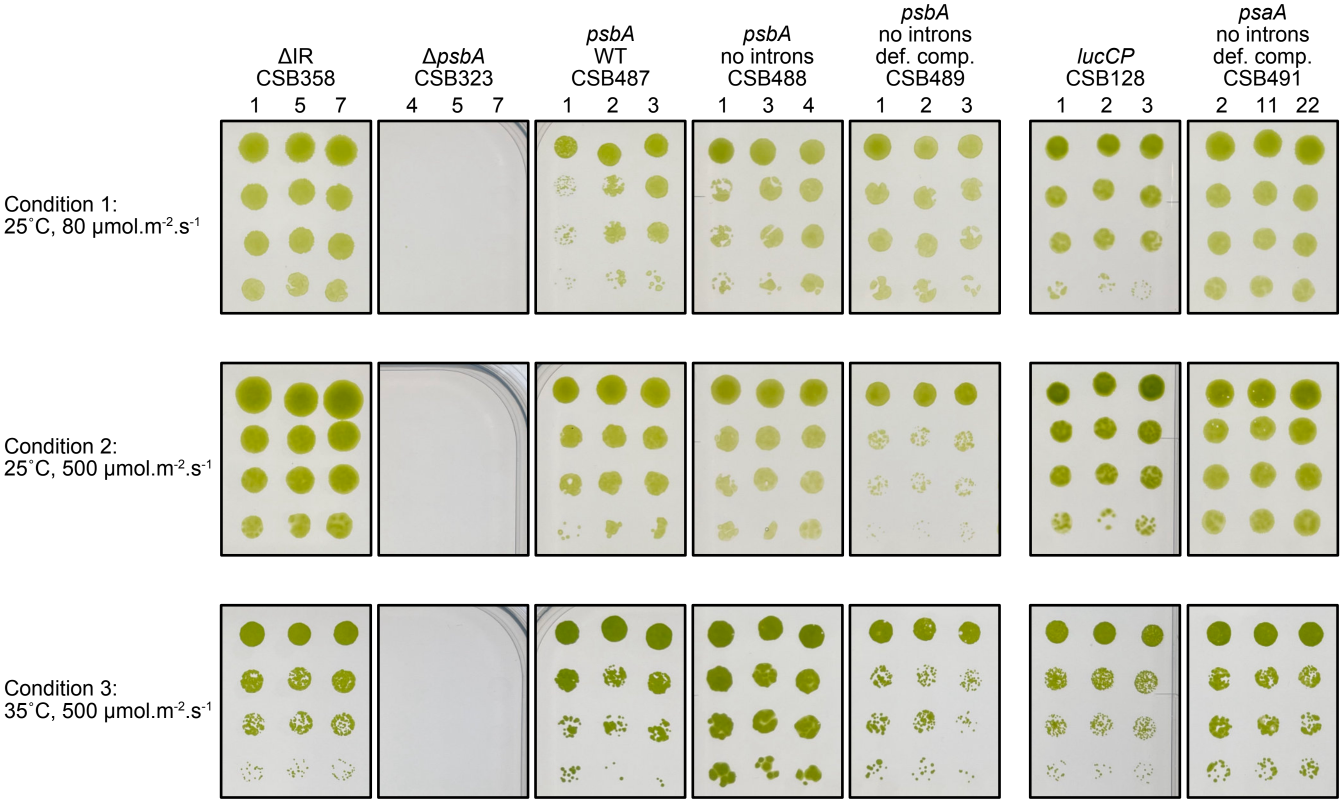

Fig. S11. Phototrophic growth of *psbA*, *psaA* and *rbcL* codon compressed strains at high light intensity and at high temperature. Cultures in the mid-logarithmic phase of growth (OD 0.5) were spotted on HSM plates and incubated under three growth conditions: 25˚C and 80 µmol.m^{-2}.s^{-1} (control), 25˚C, 500 µmol.m^{-2}.s^{-1} (high light) and 35˚C, 500 µmol.m^{-2}.s^{-1} (high light and high temperature). Dilution factors: 1, 5, 10, 100. Photographs were taken after 7 days of incubation.

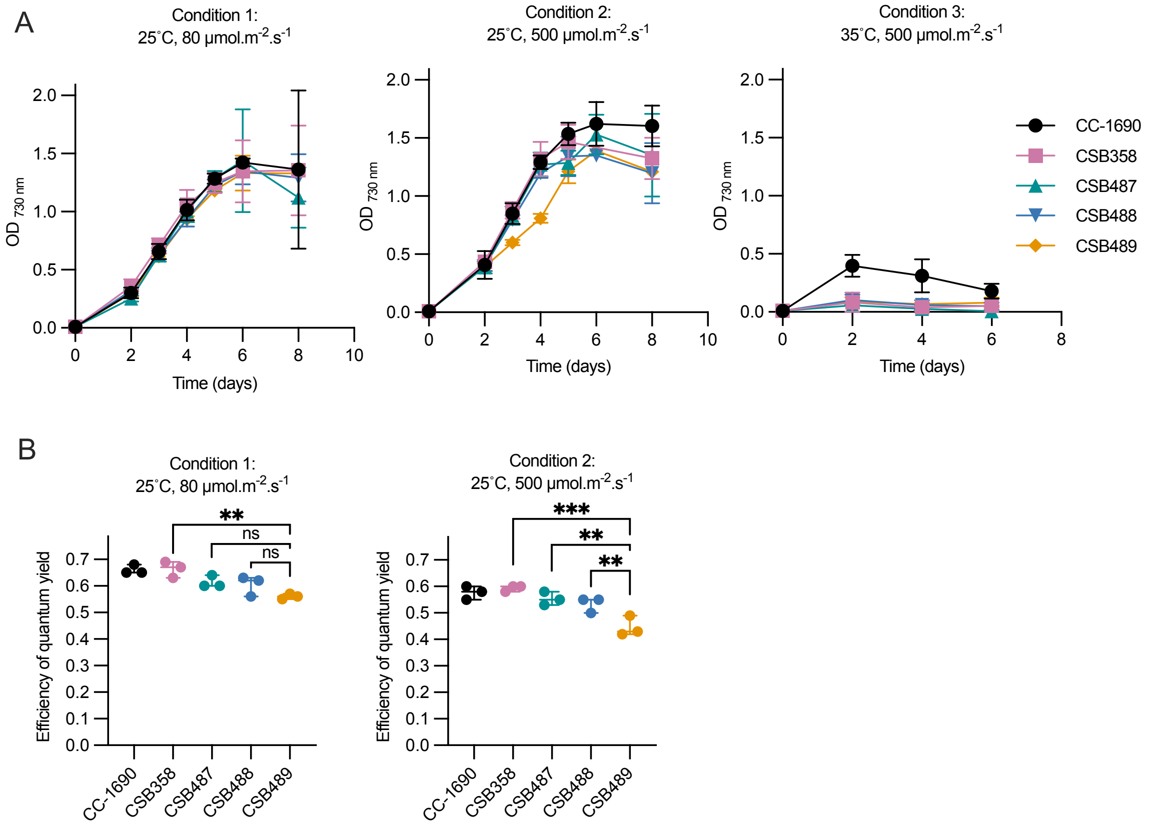

Fig. S12. Phototrophic growth and quantum efficiency of PSII of *psbA* complementation strains at high light intensity and at high temperature. (A) Phototrophic growth of homoplasmic CSB358, CSB487, CSB488 and CSB489 lines in cultures (4 mL) grown in HSM at 25˚C and light intensity of 80 µmol·m^{-2}·s^{-1} (condition 1), at 25˚C and light intensity of 500 µmol·m^{-2}·s^{-1} (condition 2), and at 35˚C and light intensity of 500 µmol·m^{-2}·s^{-1} (condition 3). Error bars represent standard deviations (n=3). **(B)** Efficiency of quantum yield of cultures grown under conditions 1 and 2 was measured on day 4. Statistical significance was determined by one-way ANOVA with Dunnett's multiple comparisons test (*p < 0.05, **p < 0.01, ***p < 0.001, ns - not significant).

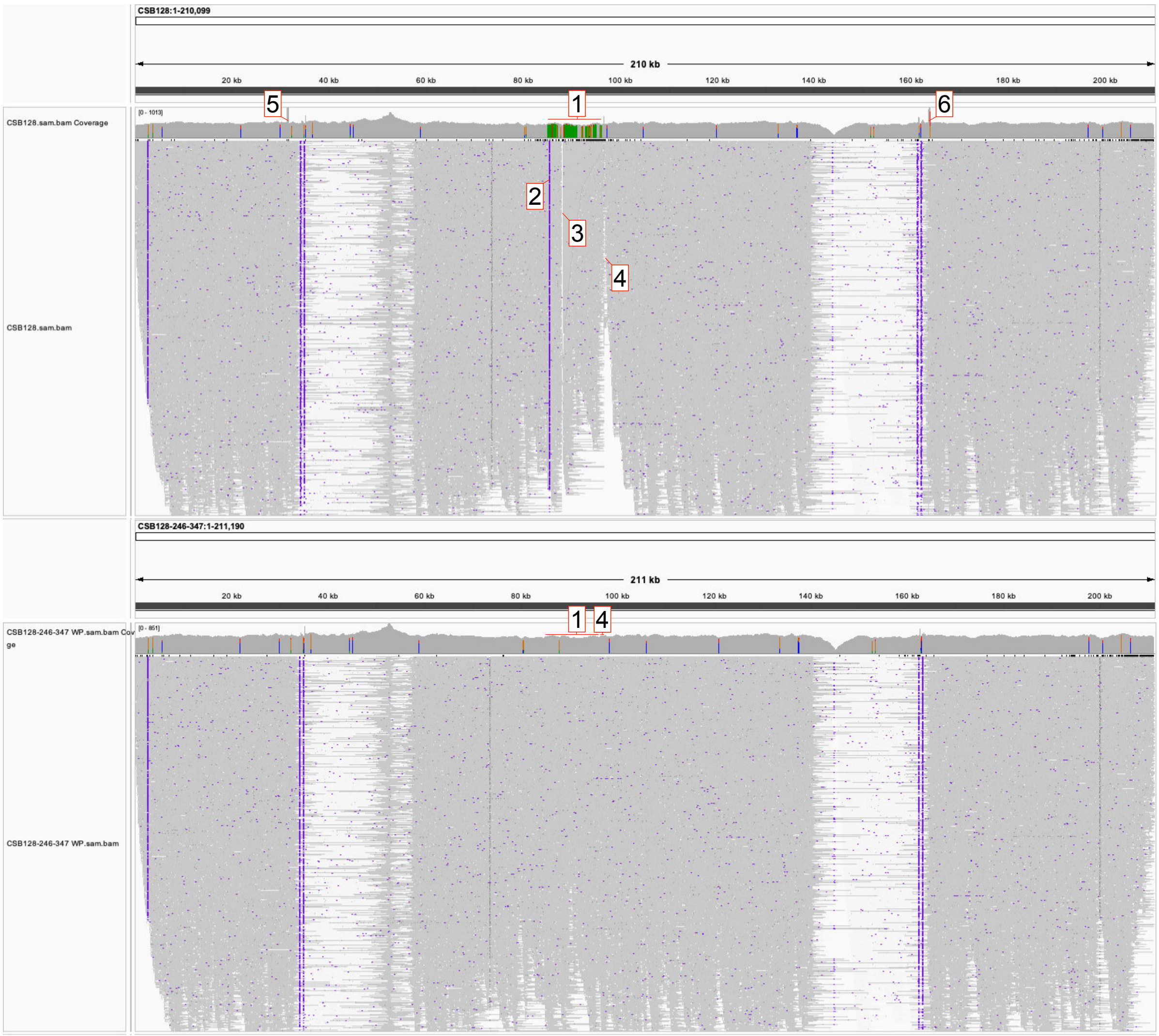

Fig. S13. ONT sequencing of CSB347-1 visualised by Integrative Genomics Viewer (IGV). Raw reads of Q score >9 were mapped using BWA-MEM to the reference genome of the parental strain CSB128 (top panel) and the expected genome sequence of CSB347. The two regions showing white at approximately 35-55 kb and 140-160 kb indicate the inverted repeats (IRs). Since the reads for the IRs map to multiple positions and have an alignment score from IGV (MAPQ) of 0, they are presented as white horizontal bars, which are not distinguishable due to high number of reads. Nonetheless, as seen in the coverage panel at the top, their coverage is consistent with the rest of the genome. Close inspection of the alignments shows the fragment of the genome encoding the *rpoA* gene and the *trnMe-psbE-rps9-ycf4-ycf3-rps18-rps2* operon have been fully recoded according to the 51 codon compressions scheme (feature 1). *rpoA* CDS has a 33 bp insertion of the FLAG sequence (feature 2), SDRs located between rpoA and rps2 are deleted (feature 3) and the *aphA6* cassette is inserted between *rpoB2* and *trnMe* (feature 4). Promoter and 5'UTR of *psaA ex1* (feature 5) and 3'UTR of *atpB* (feature 6) showed increased coverage when reads were mapped to the CSB128 reference genome, due to using them additionally for the expression of the *aphA6* marker gene. Mapping also indicated a possible heteroplasmic mutation in the intragenic space between *rpoA* and *rps2* (position 87911, expected G; A:13%, T:3%, C:8%, G:72%).

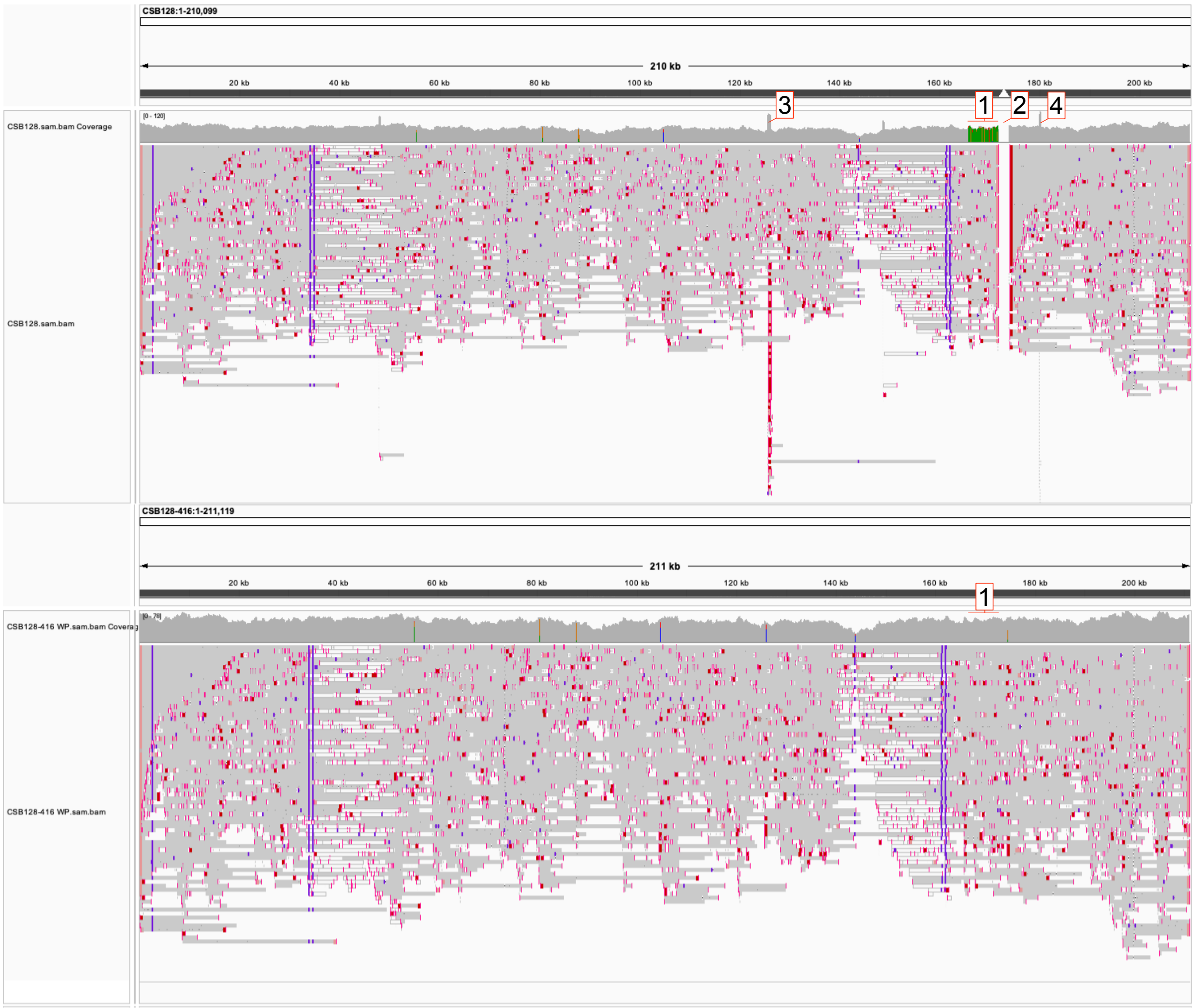

Fig. S14. ONT sequencing of CSB416-1 with a recoded *ycf1* gene. Raw reads of Q score >9 were mapped using BWA-MEM to the reference genome of the *lucCP* strain CSB128 (top panel) and the expected genome sequence of CSB416 (bottom). Analysis of the alignments shows the fragment of the genome encoding the *ycf1* coding sequence has been fully recoded according to the 51 codon compressions scheme (feature 1) and the *ycf1* promoter and 5'UTR replaced with the promoter and 5'UTR of *psbD* (feature 2). Promoter and 5'UTR of *atpA* (feature 3) and *psbD* (feature 4) showed increased coverage when reads were mapped to the CSB128 reference genome, due to using them additionally for the expression of the *codA.aadA* marker gene and *ycf1* respectively. The coverage of CSB416 is continuous and even (with exception of the inverted repeats, where reads preferentially aligned to the IR-a) indicating no large genome rearrangements.

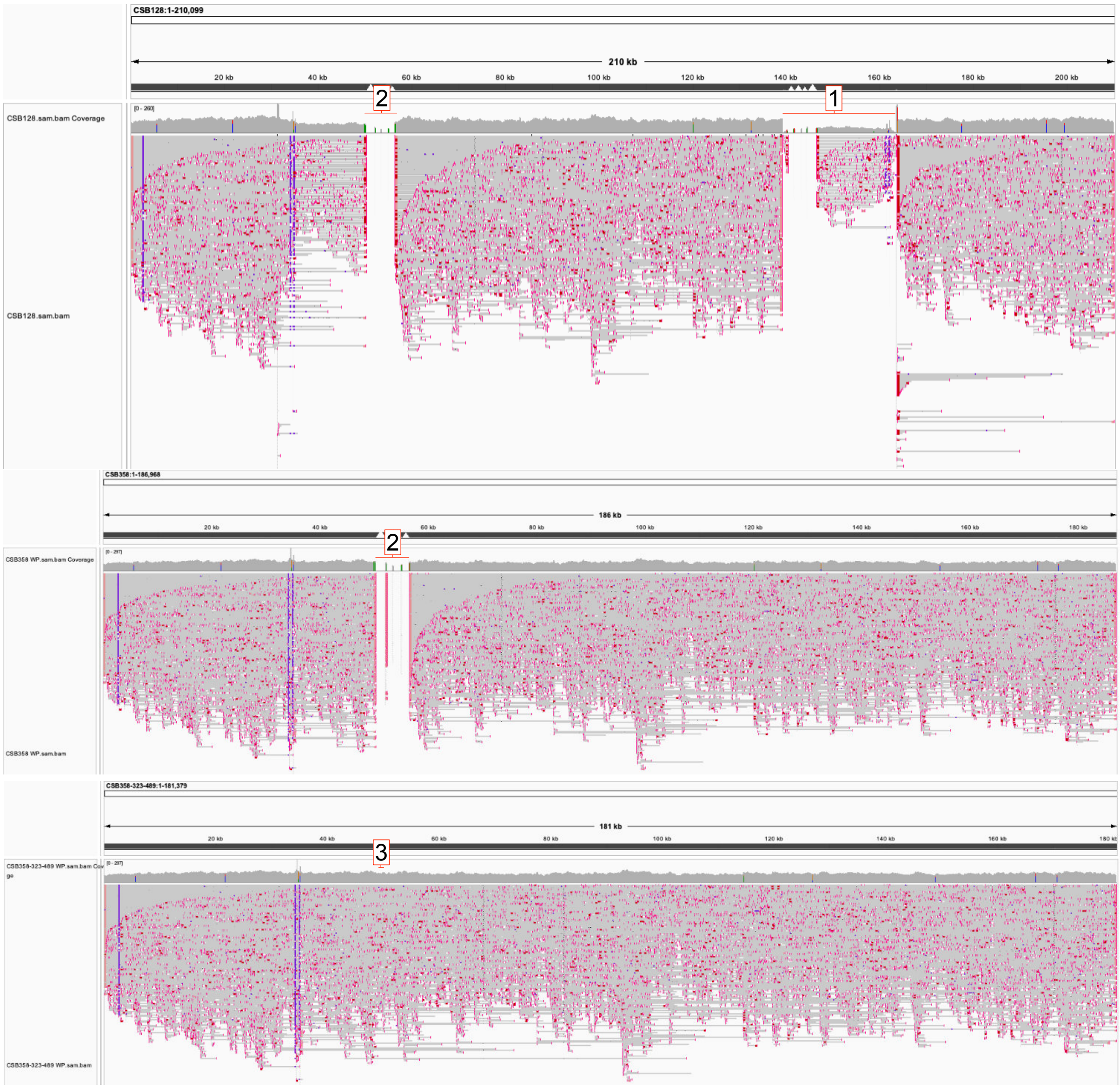

Fig. S15. ONT sequencing of CSB489-1 with a recoded *psbA* gene and its parental strains. Raw reads of Q score >9 were mapped using BWA-MEM to the reference genome of the *lucCP* strain CSB128 (top panel), the IR-deletion parental strain CSB358 (middle) and the expected genome sequence of CSB489 (bottom). Analysis of the reads shows the IR-b has been deleted (positions 139279-163681; feature 1), with some reads incorrectly aligned to IR-b, but without continuity to the *chlN* or *atpB* loci that flank the IR-b. The *psbA* gene located in IR-a lacks introns (feature 2) and the *psbA* exons are fully codon compressed (feature 3). The coverage of CSB489 is continuous and even, indicating no large genome rearrangements.

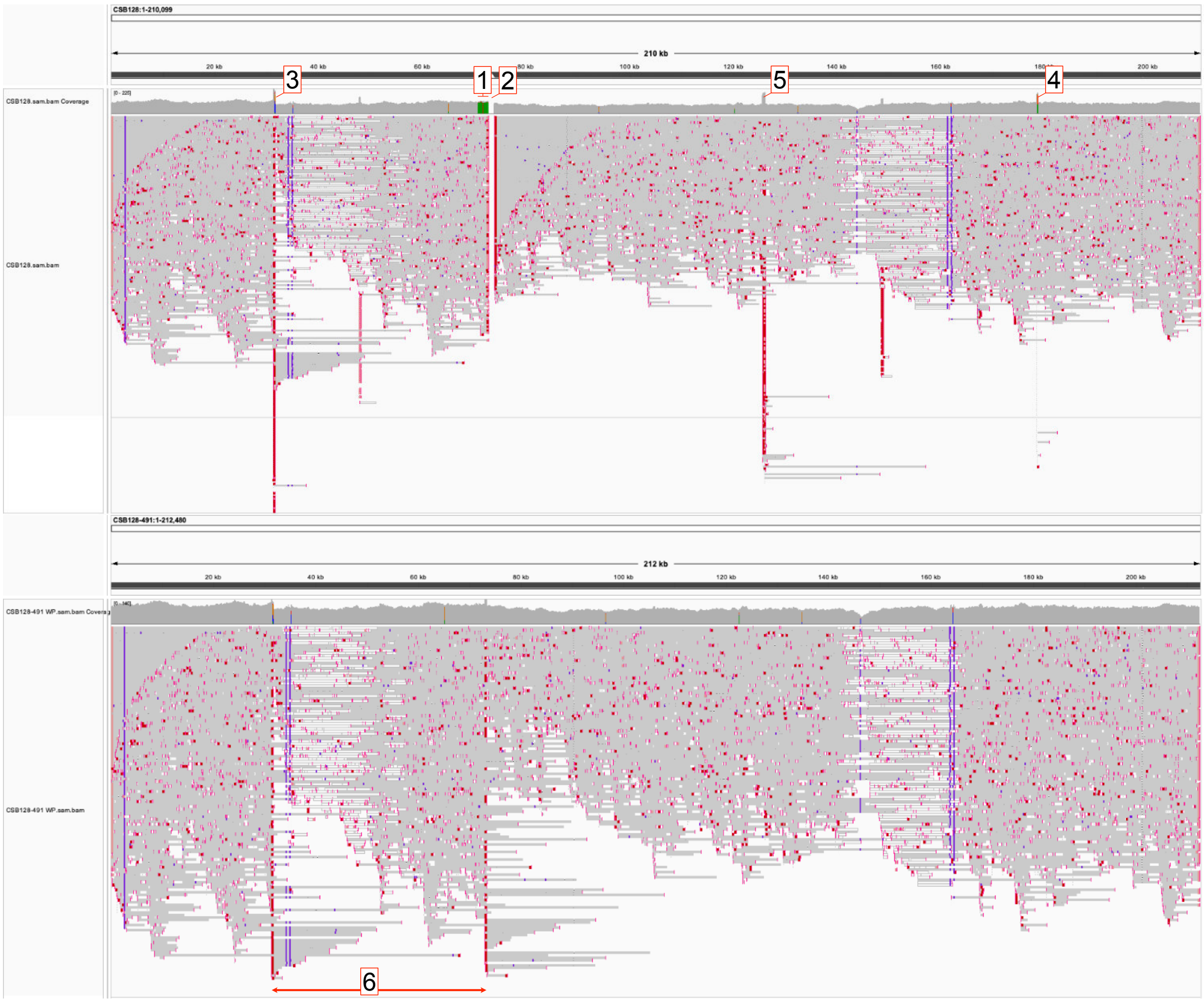

Fig. S16. ONT sequencing of CSB491-2, with a recoded complete *psaA* coding sequence in the native *psaA ex3* locus. Raw reads of Q score >9 were mapped using BWA-MEM to the reference genome of the *lucCP* strain CSB128 (top panel) and the expected genome sequence of CSB491 (bottom). Analysis of the alignments shows the fragment of the genome encoding the *psaA* coding sequence has been fully recoded according to the 51 codon compression scheme (feature 1) and the *psaA ex3* promoter and group II intron replaced with the promoter and 5’UTR of *psaA ex1* (feature 2). Reads of *psaA ex1* promoter and CDS and *psaA ex2* CDS align to the CSB128 reference sequence with doubled coverage (features 3 and 4, respectively) and represent the native WT sequences and the recoded sequences from the intron-less *psaA ex1+2+3*. Promoter and 5’UTR of *atpA* (feature 5) showed increased coverage when reads were mapped to the CSB128 reference genome, due to using it additionally for the expression of the *codA.aadA* marker gene. Inspection of the CSB491 coverage shows that some reads do not contiguously align to the reference sequences at positions 31381 and 73362 (feature 6), which could be explained by the presence of a mixed population of two genome variants, the additional one resulting from recombination between the two copies of the *psaA ex1* promoters and 5’UTR (controlling expression of the native *psaA ex1*, position 31381-31659, and the recoded intron-less *psaA ex1+2+3*, position 73362-73084) and inversion of 41.4 kb fragment of the plastome. 51% of mapped reads represent the expected genome sequence, whereas 49% of reads represent the recombined variant.

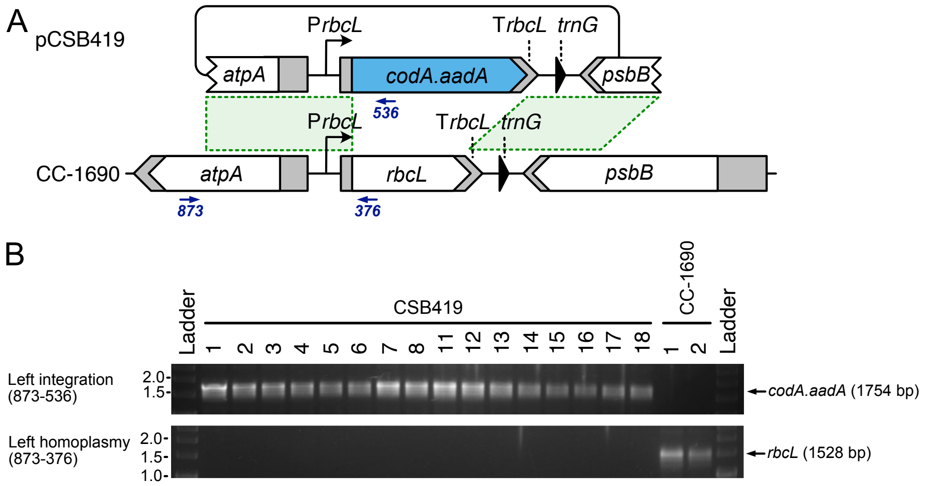

Fig. S17. Generation of the *rbcL* knockout strain. (A) Schematic showing the process of deletion of the *rbcL* gene by transformation of the plasmid pCSB419 into the strain CC-1690. The plasmid replaced a 1.4 kb fragment between the left and right homology arms (green dashed lines) with the *codA.aadA* cassette. Primers used for genotyping are represented by blue arrows. **(B)** PCR analysis of transformants confirming homoplasmic deletion of *rbcL*. Primers oligoCSB873 and oligoCSB536 were used for confirmation of the plasmid integration resulting in a product of 1754 bp. Primers oligoCSB873 and oligoCSB376 were used for the homoplasmy screen by detection of the *rbcL* CDS (product of 1528 bp).

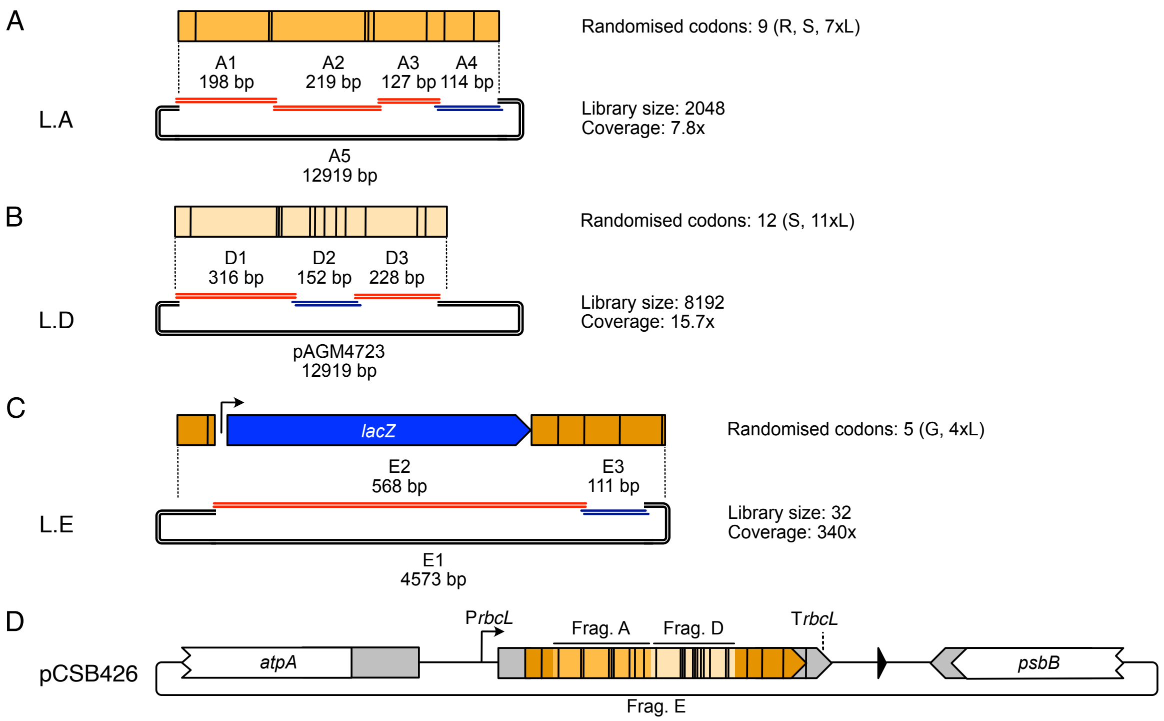

Fig. S18. Generation of the *rbcL* combinatorial library pCSB426. Plasmid library pCSB426 was constructed from three libraries of smaller sizes, L.A **(A)**, L.D **(B)** and L.E **(C)**, each encoding a different fragment of the *rbcL* coding sequence. The smaller libraries were generated by amplification of DNA fragments with degenerate nucleotides in the primer overhangs (fragments marked in red) or by annealing of degenerate single strand oligonucleotides (fragments marked in blue). Oligonucleotides used for amplification and annealing are in Table S3. Black vertical lines represent positions of the degenerate bases. Fragments were mixed, ligated by Golden-Gate reactions with BsmBI (L.A) or BbsI (L.D and L.E) and transformed to electrocompetent NEB® 10-beta *E. coli* cells. Number of randomised codons, the size of each library and the obtained coverage are indicated on the right side of each schematic. **(D)** The pCSB426 library was generated by mixing libraries L.A, L.D and L.E, ligation by Golden-Gate reaction with BsaI and transformation to electrocompetent NEB® 10-beta *E. coli* cells. Transformants were inoculated into 100 mL of LB, incubated overnight at 37°C and plasmid DNA was extracted using GenEluteTM HP Midiprep Kit (Sigma-Aldrich, UK).

3rd base	G12	R32	S76	L77	L133	L135	L138	L169	L180	L197	L219	L289	L290	L291	L314	L318	L326	L335	L343	S359	L400	L407	L424	L437	L445	L475
A	0	20	20	38	57	44	30	55	50	44	25	60	62	70	44	43	99	45	37	8	69	79	51	36	39	25
G	0	50	31	62	43	56	70	44	50	56	75	34	30	29	56	57	0	55	63	30	31	21	49	64	61	75
T	34	16	26	0	0	0	0	1	0	0	0	6	9	2	0	0	0	0	0	30	0	0	0	0	0	0
C	66	14	23	0	0	0	0	0	0	0	0	0	0	0	0	0	1	0	0	33	0	0	0	0	0	0

Fig. S19. Confirmation of degeneracy at the target codons positions in pCSB426 library. Sanger sequencing chromatograms of *E. coli* purified pCSB426 libraries were analysed using EditR 1.0.10 (6) and frequency of base at 3rd position of each target codons was calculated. The expected values were 25% of A, G, T and C for arginine and serine, 50% of T and C for glycine and 50% of A and G for leucine. Analysis showed significant degeneracy at target codon positions except for leucine 326, which was A in 99% of codons. The bases in 1st and 2nd positions did not show any degeneracy (data no shown). Data represent average frequencies for three independently assembled and sequenced pCSB426 libraries.

	G12	R32	S76	L77	L133	L135	L138	L169	L180	L197	L219	L289	L290	L291	L314	L318	L326	L335	L343	S359	L400	L407	L424	L437	L455	L475	3rd base A or T	3rd base G or C
A		18	19	42	40	35	27	49	40	30	20	52	51	54	43	32	72	30	28	13	46	51	51	32	37	21	933	
G		22	26	31	33	38	46	24	33	43	53	21	22	19	30	41	1	43	45	31	27	22	22	41	36	52		802
T	16	14	17																	14							61	
C	57	19	11																	15								102
**CSB424**	GGA	AGA	AGT	CTT	CTA	CTT	CTT	CTT	CTT	CTT	CTT	CTT	CTT	CTA	CTT	CTT	CTT	CTA	CTA	AGC	CTT	CTA	CTT	CTT	CTT	CTT		
**CSB430**	GGT	CGT	TCT	TTA	TTA	TTA	TTA	TTA	TTA	TTA	TTA	TTA	TTA	TTA	TTA	TTA	TTA	TTA	TTA	TCA	TTA	TTA	TTA	TTA	TTA	TTA	26	0
**CSB426.123**	GGT	CGC	TCG	TTA	TTA	TTA	TTA	TTG	TTA	TTA	TTG	TTA	TTA	TTA	TTA	TTG	TTA	TTA	TTA	TCA	TTA	TTA	TTA	TTA	TTA	TTA	21	5
CSB426.111	GGC	CGG	TCA	TTA	TTA	TTA	TTA	TTA	TTA	TTA	TTG	TTA	TTA	TTA	TTA	TTA	TTA	TTA	TTG	TCG	TTA	TTA	TTA	TTA	TTG	TTG	19	7
**CSB426.132**	GGT	CGT	TCG	TTA	TTA	TTG	TTA	TTA	TTA	TTA	TTA	TTA	TTA	TTA	TTG	TTG	TTA	TTG	TTA	TCT	TTA	TTG	TTA	TTA	TTA	TTG	19	7
**CSB426.137**	GGT	CGA	TCG	TTG	TTA	TTG	TTG	TTA	TTG	TTA	TTA	TTA	TTA	TTA	TTA	TTA	TTA	TTA	TTG	TCC	TTA	TTA	TTA	TTG	TTA	TTA	18	8
**CSB426.154**	GGT	CGG	TCT	TTG	TTA	TTA	TTA	TTG	TTA	TTA	TTA	TTA	TTG	TTG	TTA	TTA	TTA	TTG	TTG	TCC	TTA	TTA	TTA	TTA	TTA	TTA	18	8
CSB426.157	GGC	CGT	TCA	TTA	TTA	TTA	TTA	TTA	TTG	TTG	TTG	TTA	TTA	TTA	TTG	TTG	TTA	TTG	TTA	TCT	TTA	TTA	TTA	TTG	TTA	TTG	17	9
CSB426.26	GGC	CGT	TCC	TTA	TTA	TTA	TTA	TTA	TTG	TTA	TTG	TTA	TTA	TTA	TTG	TTA	TTA	TTG	TTG	TCG	TTA	TTA	TTA	TTA	TTA	TTG	17	9
CSB426.216	GGC	CGG	TCT	TTA	TTA	TTA	TTA	TTG	TTG	TTG	TTA	TTA	TTG	TTA	TTA	TTA	TTA	TTA	TTA	TCA	TTA	TTA	TTA	TTG	TTG	TTG	17	9
CSB426.312	GGT	CGT	TCT	TTA	TTA	TTG	TTA	TTG	TTA	TTA	TTG	TTA	TTA	TTA	TTA	TTA	TTA	TTG	TTG	TCG	TTG	TTG	TTA	TTA	TTG	TTA	17	9
CSB426.327	GGC	CGA	TCA	TTA	TTA	TTA	TTA	TTA	TTG	TTA	TTG	TTA	TTA	TTA	TTA	TTA	TTA	TTG	TTG	TCC	TTG	TTA	TTA	TTG	TTG	TTA	17	9
CSB426.17	GGC	CGG	TCA	TTA	TTG	TTG	TTG	TTA	TTG	TTG	TTG	TTA	TTA	TTA	TTA	TTA	TTA	TTA	TTA	TCA	TTA	TTG	TTA	TTA	TTA	TTG	16	10
CSB426.125	GGC	CGA	TCC	TTA	TTA	TTA	TTG	TTA	TTA	TTA	TTG	TTA	TTA	TTA	TTG	TTG	TTA	TTG	TTA	TCG	TTA	TTA	TTA	TTA	TTG	TTG	16	10
CSB426.141	GGC	CGT	TCG	TTG	TTG	TTA	TTG	TTA	TTA	TTG	TTA	TTG	TTA	TTA	TTG	TTG	TTA	TTG	TTA	TCA	TTA	TTA	TTA	TTA	TTA	TTA	16	10
CSB426.155	GGT	CGT	TCC	TTA	TTA	TTA	TTG	TTA	TTG	TTA	TTG	TTA	TTA	TTA	TTG	TTG	TTA	TTA	TTG	TCT	TTG	TTA	TTA	TTA	TTG	TTG	16	10
CSB426.21	GGT	CGG	TCA	TTA	TTG	TTG	TTG	TTA	TTG	TTG	TTG	TTA	TTA	TTA	TTG	TTA	TTA	TTG	TTA	TCA	TTG	TTA	TTA	TTA	TTA	TTA	16	10
CSB426.27	GGC	CGC	TCG	TTG	TTA	TTA	TTA	TTA	TTA	TTG	TTG	TTA	TTG	TTA	TTA	TTA	TTA	TTA	TTA	TCA	TTA	TTG	TTG	TTA	TTA	TTG	16	10
CSB426.28	GGT	CGG	TCA	TTA	TTA	TTA	TTG	TTA	TTA	TTG	TTG	TTA	TTA	TTA	TTG	TTA	TTA	TTG	TTG	TCT	TTA	TTA	TTG	TTG	TTG	TTA	16	10
CSB426.328	GGC	CGA	TCT	TTA	TTA	TTG	TTG	TTA	TTA	TTG	TTG	TTA	TTA	TTA	TTA	TTG	TTA	TTG	TTG	TCG	TTA	TTA	TTA	TTA	TTA	TTG	16	10
CSB426.14	GGC	CGA	TCA	TTA	TTA	TTG	TTG	TTG	TTG	TTA	TTA	TTG	TTA	TTA	TTA	TTG	TTA	TTG	TTG	TCG	TTA	TTA	TTA	TTA	TTA	TTG	15	11
CSB426.136	GGC	CGG	TCA	TTA	TTA	TTG	TTG	TTA	TTA	TTG	TTG	TTA	TTA	TTA	TTA	TTG	TTA	TTA	TTA	TCC	TTG	TTA	TTA	TTG	TTA	TTG	15	11
CSB426.142	GGC	CGA	TCA	TTA	TTA	TTA	TTG	TTG	TTA	TTA	TTG	TTA	TTG	TTG	TTA	TTA	TTA	TTG	TTG	TCC	TTA	TTA	TTA	TTG	TTG	TTA	15	11
CSB426.162	GGT	CGC	TCT	TTG	TTG	TTA	TTA	TTA	TTG	TTA	TTG	TTA	TTA	TTG	TTA	TTG	TTA	TTG	TTG	TCT	TTA	TTA	TTA	TTG	TTA	TTG	15	11
CSB426.212	GGC	CGC	TCT	TTA	TTA	TTG	TTG	TTA	TTA	TTG	TTA	TTG	TTA	TTA	TTA	TTG	TTA	TTG	TTG	TCG	TTA	TTA	TTA	TTA	TTA	TTG	15	11
CSB426.218	GGC	CGC	TCC	TTA	TTG	TTA	TTA	TTA	TTG	TTA	TTA	TTA	TTA	TTA	TTA	TTG	TTA	TTG	TTG	TCA	TTA	TTA	TTA	TTG	TTG	TTG	15	11
CSB426.222	GGT	CGA	TCC	TTA	TTG	TTA	TTG	TTG	TTG	TTG	TTG	TTG	TTA	TTG	TTA	TTA	TTA	TTG	TTA	TCT	TTA	TTA	TTA	TTG	TTA	TTA	15	11
CSB426.33	GGC	CGT	TCT	TTA	TTG	TTA	TTA	TTA	TTA	TTG	TTG	TTG	TTA	TTA	TTG	TTG	TTA	TTA	TTG	TCA	TTA	TTG	TTA	TTA	TTG	TTG	15	11
CSB426.35	GGC	CGA	TCG	TTG	TTA	TTG	TTA	TTA	TTA	TTA	TTG	TTA	TTA	TTA	TTA	TTG	TTA	TTG	TTG	TCT	TTA	TTA	TTG	TTG	TTA	TTG	15	11
CSB426.36	GGC	CGC	TCC	TTA	TTA	TTA	TTG	TTA	TTG	TTG	TTG	TTA	TTA	TTA	TTA	TTA	TTA	TTA	TTA	TCG	TTA	TTG	TTA	TTA	TTG	TTG	15	11
CSB426.321	GGC	CGA	TCC	TTA	TTA	TTA	TTG	TTA	TTA	TTG	TTG	TTA	TTG	TTA	TTG	TTA	TTA	TTA	TTG	TCG	TTA	TTA	TTG	TTG	TTA	TTA	15	11
**CSB426.324**	GGC	CGA	TCA	TTA	TTA	TTA	TTA	TTA	TTA	TTG	TTA	TTA	TTG	TTA	TTA	TTG	TTA	TTA	TTA	TCG	TTG	TTG	TTG	TTG	TTG	TTG	15	11
CSB426.133	GGC	CGG	TCA	TTG	TTA	TTA	TTG	TTG	TTG	TTG	TTA	TTA	TTA	TTA	TTA	TTG	TTA	TTG	TTG	TCG	TTA	TTA	TTA	TTA	TTA	TTG	14	12
CSB426.214	GGT	CGA	TCG	TTA	TTG	TTG	TTG	TTA	TTG	TTA	TTG	TTA	TTA	TTA	TTG	TTA	TTA	TTG	TTG	TCG	TTG	TTA	TTA	TTA	TTG	TTA	14	12
CSB426.221	GGC	CGA	TCA	TTA	TTA	TTG	TTG	TTA	TTA	TTG	TTG	TTA	TTA	TTG	TTA	TTA	TTA	TTG	TTG	TCG	TTA	TTA	TTG	TTG	TTG	TTA	14	12
CSB426.31	GGC	CGA	TCG	TTG	TTA	TTA	TTA	TTA	TTA	TTG	TTG	TTG	TTG	TTG	TTA	TTA	TTA	TTA	TTA	TCC	TTA	TTA	TTG	TTG	TTA	TTG	14	12
CSB426.32	GGT	CGC	TCG	TTA	TTA	TTG	TTG	TTA	TTG	TTA	TTG	TTA	TTA	TTA	TTA	TTA	TTA	TTG	TTA	TCG	TTG	TTA	TTG	TTA	TTG	TTG	14	12
CSB426.37	GGC	CGC	TCA	TTG	TTA	TTA	TTA	TTA	TTA	TTG	TTA	TTG	TTA	TTA	TTG	TTA	TTA	TTA	TTG	TCG	TTG	TTA	TTA	TTG	TTG	TTG	14	12
CSB426.15	GGC	CGC	TCA	TTA	TTG	TTG	TTA	TTA	TTG	TTG	TTG	TTG	TTG	TTA	TTA	TTG	TTA	TTA	TTA	TCC	TTG	TTA	TTA	TTA	TTA	TTG	13	13
**CSB426.127**	GGC	CGG	TCT	TTG	TTG	TTG	TTG	TTG	TTA	TTG	TTG	TTA	TTA	TTA	TTA	TTG	TTA	TTA	TTA	TCT	TTA	TTA	TTG	TTG	TTA	TTG	13	13
CSB426.134	GGC	CGC	TCA	TTG	TTG	TTG	TTA	TTA	TTG	TTA	TTG	TTA	TTA	TTA	TTG	TTA	TTA	TTG	TTG	TCT	TTA	TTG	TTA	TTG	TTA	TTG	13	13
CSB426.145	GGC	CGC	TCG	TTA	TTA	TTA	TTG	TTA	TTA	TTG	TTG	TTA	TTA	TTA	TTG	TTA	TTA	TTG	TTG	TCA	TTG	TTA	TTG	TTA	TTG	TTG	13	13
CSB426.147	GGC	CGT	TCA	TTA	TTA	TTA	TTG	TTA	TTA	TTG	TTG	TTG	TTG	TTA	TTA	TTG	TTA	TTA	TTG	TCG	TTG	TTG	TTA	TTA	TTG	TTG	13	13
CSB426.151	GGT	CGA	TCG	TTG	TTG	TTG	TTG	TTG	TTA	TTA	TTG	TTA	TTA	TTA	TTA	TTA	TTA	TTG	TTG	TCT	TTG	TTA	TTG	TTG	TTA	TTG	13	13
**CSB426.156**	GGC	CGG	TCT	TTG	TTG	TTA	TTA	TTG	TTA	TTG	TTA	TTA	TTG	TTA	TTA	TTA	TTA	TTA	TTG	TCG	TTA	TTG	TTG	TTA	TTG	TTG	13	13
CSB426.163	GGC	CGA	TCG	TTG	TTG	TTA	TTG	TTG	TTA	TTA	TTG	TTG	TTA	TTA	TTG	TTG	TTA	TTA	TTG	TCG	TTA	TTA	TTA	TTA	TTA	TTG	13	13
CSB426.215	GGT	CGC	TCG	TTA	TTG	TTG	TTG	TTG	TTA	TTA	TTG	TTA	TTA	TTA	TTA	TTG	TTA	TTG	TTG	TCG	TTA	TTA	TTA	TTG	TTG	TTA	13	13
CSB426.38	GGC	CGT	TCT	TTA	TTA	TTG	TTG	TTA	TTA	TTA	TTG	TTA	TTG	TTG	TTG	TTA	TTA	TTG	TTG	TCT	TTG	TTG	TTA	TTA	TTG	TTG	13	13
CSB426.313	GGC	CGG	TCT	TTA	TTA	TTG	TTA	TTA	TTG	TTA	TTG	TTA	TTG	TTG	TTA	TTG	TTA	TTA	TTG	TCG	TTA	TTA	TTA	TTG	TTG	TTG	13	13
CSB426.322	GGC	CGC	TCG	TTA	TTG	TTG	TTG	TTA	TTA	TTG	TTA	TTG	TTG	TTG	TTA	TTG	TTA	TTA	TTA	TCT	TTG	TTA	TTA	TTA	TTA	TTG	13	13
CSB426.334	GGC	CGG	TCA	TTG	TTA	TTA	TTA	TTA	TTG	TTG	TTG	TTA	TTA	TTA	TTG	TTG	TTA	TTG	TTG	TCC	TTA	TTG	TTA	TTG	TTA	TTA	13	13
CSB426.138	GGC	CGC	TCA	TTA	TTA	TTG	TTG	TTA	TTG	TTG	TTG	TTA	TTA	TTA	TTA	TTG	TTA	TTG	TTA	TCG	TTA	TTA	TTG	TTG	TTG	TTG	12	14
CSB426.144	GGC	CGG	TCG	TTG	TTG	TTG	TTG	TTG	TTA	TTA	TTA	TTA	TTA	TTA	TTA	TTG	TTA	TTA	TTA	TCG	TTA	TTG	TTG	TTG	TTA	TTG	12	14
CSB426.161	GGC	CGG	TCT	TTG	TTA	TTA	TTG	TTG	TTG	TTG	TTG	TTA	TTA	TTA	TTA	TTA	TTA	TTG	TTG	TCC	TTA	TTA	TTG	TTG	TTA	TTG	12	14
CSB426.23	GGC	CGT	TCG	TTG	TTG	TTG	TTG	TTA	TTA	TTG	TTG	TTA	TTA	TTA	TTG	TTG	TTA	TTG	TTA	TCC	TTG	TTA	TTA	TTA	TTA	TTG	12	14
**CSB426.323**	GGC	CGG	TCT	TTG	TTA	TTG	TTG	TTA	TTG	TTG	TTG	TTG	TTA	TTA	TTA	TTA	TTA	TTG	TTG	TCA	TTG	TTA	TTA	TTG	TTA	TTG	12	14
CSB426.124	GGC	CGC	TCT	TTG	TTG	TTG	TTG	TTA	TTG	TTA	TTG	TTG	TTA	TTA	TTA	TTG	TTA	TTA	TTA	TCC	TTA	TTG	TTA	TTG	TTG	TTG	11	15
CSB426.126	GGC	CGG	TCG	TTG	TTA	TTG	TTG	TTG	TTG	TTG	TTA	TTA	TTA	TTA	TTG	TTG	TTA	TTA	TTA	TCC	TTA	TTG	TTA	TTA	TTG	TTG	11	15
CSB426.128	GGC	CGG	TCG	TTG	TTG	TTG	TTA	TTA	TTG	TTG	TTG	TTA	TTA	TTA	TTG	TTA	TTA	TTA	TTG	TCC	TTA	TTA	TTG	TTG	TTG	TTA	11	15
CSB426.135	GGT	CGA	TCG	TTG	TTG	TTG	TTG	TTG	TTG	TTA	TTA	TTA	TTA	TTG	TTA	TTG	TTA	TTA	TTG	TCG	TTG	TTA	TTG	TTG	TTA	TTG	11	15
CSB426.211	GGC	CGT	TCG	TTG	TTG	TTG	TTA	TTA	TTA	TTG	TTG	TTA	TTA	TTG	TTG	TTG	TTA	TTA	TTG	TCA	TTG	TTA	TTA	TTG	TTG	TTG	11	15
CSB426.333	GGC	CGG	TCG	TTG	TTA	TTG	TTA	TTA	TTA	TTG	TTG	TTA	TTG	TTG	TTG	TTA	TTA	TTG	TTG	TCG	TTA	TTA	TTA	TTG	TTG	TTA	11	15
CSB426.335	GGT	CGG	TCG	TTG	TTG	TTA	TTG	TTA	TTG	TTG	TTA	TTG	TTG	TTG	TTA	TTG	TTA	TTG	TTA	TCT	TTA	TTA	TTA	TTG	TTG	TTG	11	15
CSB426.12	GGC	CGG	TCG	TTG	TTG	TTG	TTG	TTA	TTA	TTG	TTG	TTG	TTA	TTG	TTG	TTG	TTA	TTA	TTA	TCA	TTG	TTG	TTA	TTA	TTG	TTA	10	16
CSB426.131	GGC	CGG	TCG	TTG	TTG	TTG	TTA	TTA	TTG	TTG	TTA	TTG	TTA	TTA	TTG	TTA	TTA	TTG	TTG	TCG	TTG	TTA	TTA	TTG	TTA	TTG	10	16
CSB426.143	GGC	CGA	TCT	TTA	TTA	TTG	TTG	TTA	TTA	TTG	TTA	TTG	TTG	TTG	TTG	TTG	TTA	TTG	TTG	TCG	TTG	TTA	TTG	TTG	TTA	TTG	10	16
**CSB426.148**	GGC	CGT	TCC	TTA	TTG	TTA	TTA	TTG	TTG	TTG	TTA	TTG	TTG	TTG	TTA	TTG	TTG	TTG	TTG	TCT	TTG	TTG	TTA	TTG	TTA	TTA	10	16
CSB426.164	GGC	CGC	TCC	TTA	TTG	TTA	TTG	TTG	TTA	TTG	TTG	TTA	TTG	TTA	TTG	TTG	TTA	TTG	TTG	TCA	TTA	TTA	TTG	TTA	TTG	TTG	10	16
CSB426.24	GGC	CGT	TCC	TTA	TTA	TTG	TTG	TTG	TTA	TTG	TTG	TTA	TTG	TTG	TTA	TTG	TTA	TTA	TTG	TCG	TTA	TTG	TTA	TTG	TTG	TTG	10	16
CSB426.217	GGC	CGT	TCT	TTA	TTG	TTG	TTG	TTG	TTA	TTA	TTG	TTG	TTA	TTA	TTA	TTG	TTA	TTG	TTG	TCC	TTG	TTG	TTG	TTA	TTG	TTG	10	16
CSB426.34	GGC	CGA	TCG	TTG	TTG	TTG	TTG	TTG	TTA	TTG	TTG	TTA	TTG	TTG	TTG	TTA	TTA	TTA	TTA	TCG	TTA	TTG	TTG	TTG	TTA	TTA	10	16
**CSB426.331**	GGC	CGG	TCG	TTG	TTG	TTA	TTG	TTA	TTA	TTA	TTG	TTA	TTA	TTG	TTG	TTA	TTG	TTG	TTG	TCG	TTA	TTA	TTG	TTG	TTG	TTG	10	16
**CSB426.326**	GGC	CGC	TCA	TTA	TTG	TTA	TTG	TTG	TTG	TTG	TTG	TTG	TTA	TTA	TTA	TTG	TTA	TTG	TTA	TCG	TTG	TTG	TTA	TTG	TTG	TTG	9	17
**CSB426.332**	GGC	CGC	TCT	TTG	TTG	TTG	TTG	TTG	TTG	TTA	TTG	TTA	TTG	TTA	TTG	TTG	TTA	TTG	TTG	TCC	TTG	TTA	TTA	TTG	TTA	TTA	9	17
**CSB426.158**	GGC	CGC	TCC	TTA	TTG	TTA	TTG	TTA	TTG	TTA	TTG	TTG	TTG	TTG	TTG	TTA	TTA	TTG	TTA	TCG	TTG	TTG	TTA	TTG	TTG	TTG	8	18

Fig. S20. Summary of the codon compressed *rbcL* coding sequences. Composition of the 26 target codons in 73 transformants selected from the CSB426 library are compared to those in the WT *rbcL* (CSB424) and the *rbcL* compressed using the defined compression scheme (CSB430). Sequences are sorted based on the decreasing number of codons ending with A or T. Randomised codons are coloured by the third base of the codon: T - red, C - blue, A - green, G - black. Counts of codons are presented at the top of the figure. Sequences highlighted in yellow were selected for subsequent analysis.

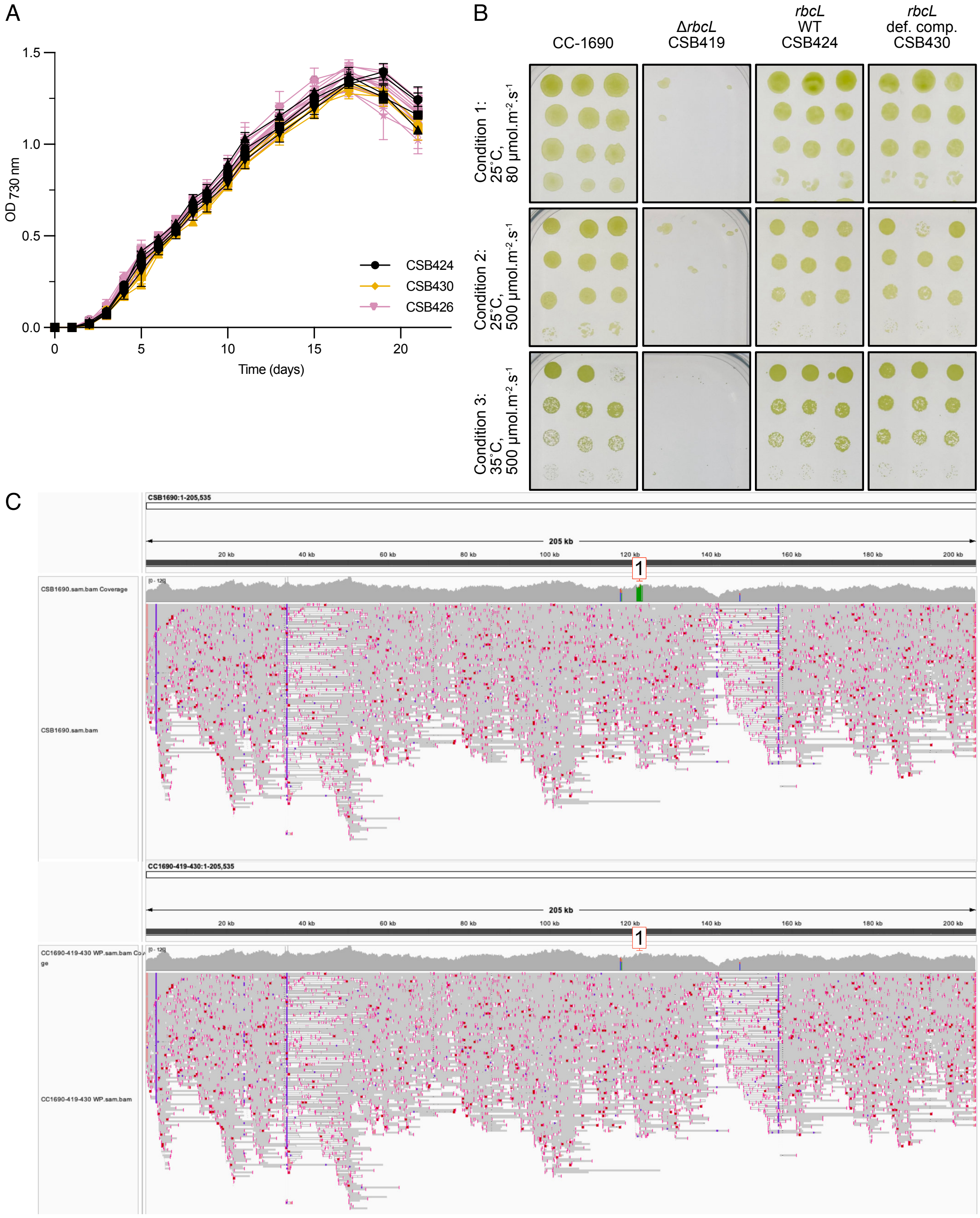

Fig. S21. Characterisation of the *rbcL* codon compressed strains (A) Phototrophic growth of the *rbcL* complemented lines. Selected transformants of pCSB424 (black; n=4), pCSB430 (orange; n=4) and pCSB426 (pink; n=13) were grown in HSM at light intensity of 80 µmol·m^{-2}·s^{-1}. OD_{730nm} of cultures was measured every 24-48 h. Error bars represent standard deviations for three independent cultures. **(B)** Phototrophic growth of CSB424 and CSB430 at high light intensity and at high temperature. **(C)** ONT sequencing of CSB430-22. Raw reads of Q score >9 were mapped using BWA-MEM to the reference genome of the WT strain CC-1690 (top panel) and the expected genome sequence of CSB430 (bottom). Analysis of the alignments shows the fragment of the genome encoding the *rbcL* coding sequence has been fully recoded according to the 51 codon compressions scheme (feature 1). The coverage of CSB430-22 is continuous and even (with exception of the inverted repeats, where reads preferentially aligned to the IR-a), indicating no large genome rearrangements.

Table S1. List of plasmids generated and used in this study. For level 2 plasmids assembled by Golden Gate cloning, individual level 1 plasmids are highlighted with alternating highlighting. Sequences are available in the GenBank database (www.ncbi.nlm.nih.gov/genbank/) with the appropriate accession numbers.

Plasmid	Backbone	Level	Description	Accession number
pPM900	-	-	Level 2 recipient plasmid (XY)	MT361981
pCSB19	-	-	Level 2 recipient plasmid (AZ)	PP746763
pCSB245	pPM900	2	LHA(rpoA)-PatpA-5'atpA-codA.aadA-TrbcL-PpsbD-5'psbD-rpoA_FLAG(WT)-RHA(rpoA)	PV101188
pCSB246	pPM900	2	LHA(rpoA)-PatpA-5'atpA-codA.aadA-TrbcL-PpsbD-5'psbD-rpoA_FLAG(Def.comp.)-RHA(rpoA)	PV101189
pCSB247	pPM900	2	LHA(rpoA)-PatpA-5'atpA-codA.aadA-TrbcL-PpsbD-5'psbD-rpoA_FLAG(ChimeraMAP)-RHA(rpoA)	PV101190
pCSB248	pPM900	2	LHA(rpoA)-PatpA-5'atpA-codA.aadA-TrbcL-PpsbD-5'psbD-rpoA_FLAG(CUO)-RHA(rpoA)	PV101191
pCSB317	pPM900	2	LHA(ycf1)-PatpA-5'atpA-codA.aadA-TrbcL-PpsbD-5'psbD-ycf1(WT)-RHA(ycf1)	PV101193
pCSB322	pPM900	2	LHA(rpoA)-PpsaA-5'psaA-aphA6-TatpB-PrpoA-5'rpoA-rpoA_FLAG(Def.comp.)	PV101192
pCSB323	pPM900	2	LHA(psbA)-PatpA-5'atpA-codA.aadA-TrbcL-RHA(psbA)	PV101195
pCSB347	pPM900	2	LHA(rpoB2)-PpsaA-5'psaA-aphA6-TatpB-psbE-rps2 operon(Def.comp)-PrpoA-5'rpoA-rpoA(Def.comp)	PV101200
pCSB416	pPM900	2	LHA(ycf1)-PatpA-5'atpA-codA.aadA-TrbcL-PpsbD-5'psbD-ycf1(Def.comp.)-RHA(ycf1)	PV101194
pCSB419	pCSB19	2	LHA(rbcL)-PrbcL-5'rbcL-codA.aadA-TrbcL-RHA(rbcL)	PV101201
pCSB424	pCSB19	2	LHA(rbcL)-PrbcL-5'rbcL-rbcL(WT)-TrbcL-RHA(rbcL)	PV101202
pCSB430	pCSB19	2	LHA(rbcL)-PrbcL-5'rbcL-rbcL(Def.comp.)-TrbcL-RHA(rbcL)	PV101203
pCSB426 library	pCSB19	2	LHA(rbcL)-PrbcL-5'rbcL-rbcL(Combinatorial library)-TrbcL-RHA(rbcL)	PV101204
pCSB487	pCSB19	2	LHA(psbA)-PpsbA-psbA-TpsbA-RHA(psbA)	PV101196
pCSB488	pCSB19	2	LHA(psbA)-PpsbA-psbA(no-introns)-TpsbA-RHA(psbA)	PV101197
pCSB489	pCSB19	2	LHA(psbA)-PpsbA-psbA(no-introns,Def.comp.)-TpsbA-RHA(psbA)	PV101198
pCSB491	pPM900	2	LHA(psaA.ex3)-PatpA-5'atpA-codA.aadA-TrbcL-PpsaA.ex1-5'psaA.ex1-psaA.ex1+2+3(Def.comp.)-RHA(TpsaA.ex3)	PV101199

Table S2. List of *Chlamydomonas reinhardtii* strains used or generated in this study. Transgenic strains from this study were generated by the introduction of the relevant plasmids detailed in Table S1.

Strain	Genetic background	Description	Reference
CC-1690	-	WT strain (21 gr)	(7)
CSB128 (Luc1.R2)	CC-1690	WT CC-1690 containing *lucCP* firefly luciferase cassette in the *rrn5-psbA* insertion site	(8)
CSB128-358	CSB128	Transformed with plasmid pCSB358. Inverted repeat replaced with *aphA6* cassette.	This work
CSB245<br>CSB246<br>CSB247<br>CSB248	CSB128	Transformed with plasmids pCSB245 (WT *rpoA* CDS), pCSB246 (Def. comp.), pCSB247 (ChimeraMAP) and pCSB248 (CUO), resulting in recoded rpoA CDS, *rpoA* promoter and 5'UTR swapped for those of *psbD,* deletion of SDRs, and integration of the FLAG-tag	This work
CSB322	CSB246	CSB246-1 transformed with pCSB322. *rpoA* promoter and 5'UTR restored upstream of *rpoA* CDS	This work
CSB317<br>CSB416	CS128	Transformed with plasmids pCSB317 (WT *ycf1* CDS) and pCSB416 (*ycf1* Def. comp.), resulting in recoded *ycf1* CDS, *ycf1* promoter and 5'UTR swapped for those of *psbD*	This work
CSB323	CSB128-358	Transformed with plasmid pCSB323, *psbA* gene replaced with the *codA.aadA* cassette. Non-photosynthetic phenotype.	This work
CSB487<br>CSB488<br>CSB489	CSB323	*psbA* knockout complemented with pCSB487 (*psbA* WT), pCSB488 (*psbA* WT, no introns) or pCSB489 (*psbA* Def. comp., no introns)	This work
CSB491	CSB128	Transformed with pCSB491, *psaA* made intron-less and recoded using Def. comp. scheme	This work
CSB347	CSB246	CSB246-1 transformed with pCSB347. *trnM*^{e}*-rps2* operon recoded according to the Def. comp. scheme.	This work
CSB419	CC-1690	Transformed with plasmid pCSB419, *rbcL* CDS replaced with *codA.aadA*. Non-photosynthetic and light sensitive phenotype.	This work
CSB424<br>CSB426<br>CSB430	CSB419	*rbcL* knockout complemented with pCSB424 (*rbcL* WT), pCSB430 (*rbcL* Def. comp.) or pCSB426 library (combinatorial assembly of codons for target positions)	This work

Table S3. List of synthetic oligonucleotides used in this study.

Name	Sequence	Description
**Genotyping of CSB245, CSB246, CSB247 and CSB248 transformants**		
oligoCSB534	TGGTTTCAATTATTAAAGGTCAAGG	*rpoA* locus F
oligoCSB535	CTCCCTCGTACAAAGCATAC	*rpoA* locus R
oligoCSB536	CGTGTTGAATACCGTTAGCA	*codA.aadA* cassette R
**Amplification of *rpoA* CDS in CSB246 and CSB248, and Sanger sequencing**		
oligoCSB145	GCGCAGATCAGTTGGAAGA	*rpoA* amplification F
oligoCSB459	TGAAGGTTTTTGGTGGAATTG	*rpoA* sequencing
oligoCSB471	TCAACAAATATGAATTATCGAGCAA	*rpoA* sequencing
oligoCSB501	TTTTAGTTGCTCAAAGGGGTTT	*rpoA* sequencing
oligoCSB537	TGCCAGTTTCCTCCTTTCTC	*rpoA* amplification R
**Genotyping of CSB322 transformants**		
oligoCSB95	GTTCTTAGAGCTAAAAGAGAAGAACAA	*codA.aadA* cassette R
oligoCSB534	TGGTTTCAATTATTAAAGGTCAAGG	*rpoA* locus F
oligoCSB535	CTCCCTCGTACAAAGCATAC	*rpoA* locus R
oligoCSB611	CTTCACGAGAAACTGAGTATG	*aphA6* cassette R
**Amplification of *rpoA* in CSB322 for Amplicon-NGS**		
oligoCSB713	ACACTCTTTCCCTACACGACGCTCTTCCGATCTATGAC AATTTATCCAAATTTAAAAAAAATC	*rpoA* product 1 F
oligoCSB714	GACTGGAGTTCAGACGTGTGCTCTTCCGATCTATTTCA TATTTAAAAAACCATCCTC	*rpoA* product 1 R
oligoCSB715	ACACTCTTTCCCTACACGACGCTCTTCCGATCTCAACA TTAGCTGAGGATGG	*rpoA* product 2 F
oligoCSB716	GACTGGAGTTCAGACGTGTGCTCTTCCGATCTGCTTTG TTAACGAATTTATGC	*rpoA* product 2 R
oligoCSB717	ACACTCTTTCCCTACACGACGCTCTTCCGATCTCTGAC CCATCTTTAGAGTTTTC	*rpoA* product 3 F
oligoCSB718	GACTGGAGTTCAGACGTGTGCTCTTCCGATCTCCTAAA AAAGTTTTAATTTTTGTTAATG	*rpoA* product 3 R
oligoCSB719	ACACTCTTTCCCTACACGACGCTCTTCCGATCTTCAAC AGGCATTTCAAATACTG	*rpoA* product 4 F
oligoCSB720	GACTGGAGTTCAGACGTGTGCTCTTCCGATCTAGATTA ATGCTTGATATAAAGCTTTG	*rpoA* product 4 R
oligoCSB721	ACACTCTTTCCCTACACGACGCTCTTCCGATCTTCCAC GCAAAGCTTTATATC	*rpoA* product 5 F
oligoCSB722	GACTGGAGTTCAGACGTGTGCTCTTCCGATCTATCAGT AACTAAAGTTAAGCCTAAC	*rpoA* product 5 R
**Genotyping of CSB317 and CSB416 transformants**		
oligoCSB95	GTTCTTAGAGCTAAAAGAGAAGAACAA	*codA.aadA* cassette R
oligoCSB219	TCAACAGCACCTGTAATTGCTA	*ycf1* locus F
oligoCSB711	TAGCTTGTCACTGCCATTCC	*ycf1* promoter F
oligoCSB712	ACTGCAAACGCAAAACATTG	*ycf1* promoter R
**Amplification of ycf1 in CSB416 and Amplicon-NGS**		
oligoCSB921	ATTCAATTGAAGAAGATGATTTAC	*ycf1* product d F
oligoCSB922	CTTGTACCACTTTAGTTAATTG	*ycf1* product d R
oligoCSB923	AGCTACACGTAAACCACG	*ycf1* product c F
oligoCSB924	GTATTTGATAATGGTTTAAATGAAAC	*ycf1* product c R
oligoCSB929	GAATTTGGTGCGGTTTTTAC	*ycf1* product a F
oligoCSB930	CCATGGGATTACAAAAAAACG	*ycf1* product a R
oligoCSB931	GTAGCTTCATTACGTCGTG	*ycf1* product b F
oligoCSB932	TGTATCATAATGTTCCGATAATAAG	*ycf1* product b R
**Genotyping of CSB323 transformants**		

oligoCSB37	CAATGACAGAAAAAGAAATTGTTGA	*lucCP* CDS R
oligoCSB95	GTTCTTAGAGCTAAAAGAGAAGAACAA	*codA.aadA* CDS R
oligoCSB591	GGTAGGTTCTGTCACTGAC	*psaA* 3'UTR F
oligoCSB728	GGGAAGAGAATGGGTTCGAT	*psbA* left flank F
oligoCSB731	GGCTAGAATTTTCACGACGTTC	*psbA ex 1* CDS R
**Genotyping of CSB487, CSB488 and CSB489 transformants**		
oligoCSB95	GTTCTTAGAGCTAAAAGAGAAGAACAA	*codA.aadA* CDS R
oligoCSB728	GGGAAGAGAATGGGTTCGAT	*psbA* left flank F
oligoCSB1020	TGGTAAGGACCACCGTTGTA	*psbA ex 2* CDS R
**Genotyping of CSB491 transformants**		
oligoCSB95	GTTCTTAGAGCTAAAAGAGAAGAACAA	*codA.aadA* CDS R
oligoCSB1104	TAACCTGTCGCAGGCCATTA	*wendy2* F
oligoCSB1105	CCTTGGAAACCACCACCTAC	*psaA ex 3* CDS R
oligoCSB1106	CGGCACGTAGTTGGAAAGTA	*psaA ex 3* g. II intron F
**Genotyping of CSB347 transformants**		
oligoCSB95	GTTCTTAGAGCTAAAAGAGAAGAACAA	*codA.aadA* CDS R
oligoCSB534	TGGTTTCAATTATTAAAGGTCAAGG	*rpoA* locus F
oligoCSB535	CTCCCTCGTACAAAGCATAC	*rpoA* locus R
oligoCSB611	CTTCACGAGAAACTGAGTATG	*aphA6* cassette R
oligoCSB629	CGGACGACATGGGAATAAAG	*rpoB2* CDS F
oligoCSB738	GCCTCTTACTCCCAGATATCCA	P*trnMe* R
**Amplification of *trnM^{e}-rps2* operon in CSB416 and Amplicon-NGS**		
oligoCSB815	ACACTCTTTCCCTACACGACGCTCTTCCGATCTCTATG TTAAACAAAAAGCCAC	product 1 F
oligoCSB816	GACTGGAGTTCAGACGTGTGCTCTTCCGATCTTTTCAC CAATTGTAATAGCAG	product 1 R
oligoCSB817	ACACTCTTTCCCTACACGACGCTCTTCCGATCTTAATT TAGCAGCATTAACACCAG	product 2 F
oligoCSB818	GACTGGAGTTCAGACGTGTGCTCTTCCGATCTGTTAAA TTGCCTGCTGAAC	product 2 R
oligoCSB821	ACACTCTTTCCCTACACGACGCTCTTCCGATCTCAAAA GTTGAAGCACGTC	product 4 F
oligoCSB822	GACTGGAGTTCAGACGTGTGCTCTTCCGATCTTACCAG ACGTTGAGTTTG	product 4 R
oligoCSB823	ACACTCTTTCCCTACACGACGCTCTTCCGATCTGAAAA ACGTCAAACAATTAAAG	product 5 F
oligoCSB824	GACTGGAGTTCAGACGTGTGCTCTTCCGATCTAACCAT ATATAAATTTTGATTGTCTTG	product 5 R
oligoCSB825	ACACTCTTTCCCTACACGACGCTCTTCCGATCTAAATT ACGTGAGTTAAAAGC	product 6 F
oligoCSB826	GACTGGAGTTCAGACGTGTGCTCTTCCGATCTTTTAAT TTGACGTAATTCAGCAAC	product 6 R
oligoCSB827	ACACTCTTTCCCTACACGACGCTCTTCCGATCTAGTTA AAACTAAAATGAACGTTTATG	product 7 F
oligoCSB828	GACTGGAGTTCAGACGTGTGCTCTTCCGATCTCATTCA CGTACAGCATTC	product 7 R
oligoCSB835	ACACTCTTTCCCTACACGACGCTCTTCCGATCTCGGTA GGTTTAGATACACG	product 3 F
oligoCSB836	GACTGGAGTTCAGACGTGTGCTCTTCCGATCTTAGTTA ATTGTGCTAAAGCTTTAG	product 3 R
oligoCSB837	ACACTCTTTCCCTACACGACGCTCTTCCGATCTCTAAA ATGAAATATCCTATGGATTC	product 8 F

oligoCSB838	GACTGGAGTTCAGACGTGTGCTCTTCCGATCTGACGTC CTAGAATGACAAC	product 8 R
oligoCSB875	AAAAGCTAAGTAAGCACAC	product 9 F
oligoCSB876	GCTAACTGACATCAATGGAC	product 9 R
oligoCSB877	TAGCAAAAGATGAAAAAATCTCTCA	product 10 F
oligoCSB878	CAGTTTGGCCCATTAAAC	product 10 R
oligoCSB879	CACAAATTTAATGCAATTTTCAG	product 11 F
oligoCSB880	GCTAACTATTACACTTCTTTGG	product 11 R
oligoCSB881	AACTCAATCAATTCTATATGACAC	product 12 F
oligoCSB882	CAACACGAATAGCTTCAATATC	product 12 R
oligoCSB883	TTGTTCCGTTTTTCCCAC	product 13 F
oligoCSB884	TAATAACATCTGTTTAGACGTTTTG	product 13 R
oligoCSB887	TGTCAGCACAAGCTGAAG	product 15 F
oligoCSB888	CTTAGAGAAGAGTAAAAATCAATCTG	product 15 R
oligoCSB895	TTTATTTATTTATGCGCGGTTTAAAATC	product 16 F
oligoCSB896	CAAGTTTTTATAACACTGGATTTG	product 16 R
oligoCSB897	TCGCTGATTTTATTTTTAATGC	product 14 F
oligoCSB899	GCTTCTTTCCAGTAATCTGC	product 14 R
**Genotyping of CSB419**		
oligoCSB376	TGGAGTCATACGGAATGCAG	*rbcL* CSD R
oligoCSB536	CGTGTTGAATACCGTTAGCA	*codA.aadA* CDS R
oligoCSB873	GCGATTGCTGTTTTACCTGTT	*atpA* CDS F
**Generation of pCSB426 library**		
oligoCSB849	GTGTTGCGTCTCATGCGTGAGACCCACGAAGTGATCCG TTTAAAC	fragment A5 F
oligoCSB850	CAGTGTCGTCTCAATGTTGAGACCCAGAGTGTTCAACC CCAG	fragment A5 R
oligoCSB851	GTGTTGCGTCTCAACATACTACACACCTGATTACGTAG TACGNGATACTGATATTTTAGCTGCATTC	fragment A1 F
oligoCSB852	CAGTGTCGTCTCAAACGGTCYAANGATGTTAAACCGTC AGTCCATAC	fragment A1 R
oligoCSB853	GTGTTGCGTCTCACGTTACAAAGGTCGTTGTTAC	fragment A2 F
oligoCSB854	CAGTGTCGTCTCACAGGTGGAATACGYAAGTCTTCYAA ACGYAAAGCACGTAAAGCTTTGAAAC	fragment A2 R
oligoCSB855	GTGTTGCGTCTCACCTGCTTACGTTAAAACATTC	fragment A3 F
oligoCSB856	CAGTGTCGTCTCATTGATTGTACAACCTAAYAAACCAC GACCATATTTGTTTAATTTG	fragment A3 R
oligoCSB857	TCAAACCTAAATTAGGTTTRTCAGCTAAAAACTACGGT CGTGCAGTTTATGAATGTTTACGTGGTGGTTTRGACTT TACTAAAGACGACGAAAACGTAAACTCACAACCATTCA	fragment A4 sense
oligoCSB858	CGCATGAATGGTTGTGAGTTTACGTTTTCGTCGTCTTT AGTAAAGTCYAAACCACCACGTAAACATTCATAAACTG CACGACCGTAGTTTTTAGCTGAYAAACCTAATTTAGGT	fragment A4 antisense
oligoCSB859	AAGGGGTTGAAGACAATGCCGGTCTCATGCGTTGGCGT GACCGTTTCTTRTTCGTTGCTGAAGCTATTTAC	fragment D1 F
oligoCSB860	CAGTGTTGGAAGACAAGGTCAATAACCGCGTGCATAGC ACGGTGGATGTGYAAYAAYAAACCGTTGTCACGACAG	fragment D1 R
oligoCSB861	GACCGTCAACGTAACCACGGTATTCACTTCCGTGTTTT RGCTAAAGCTTTRCGTATGTCTGGTGGTGACCACTTRC ACTCTGGTACTGTTGTAGGTAAATTRGAAGGTGAACGT GAAGTTACTTTRGGTTTCGTAGACTTAATGCGTGATGA	fragment D2 sense
oligoCSB862	GTAGTCATCACGCATTAAGTCTACGAAACCYAAAGTAA CTTCACGTTCACCTTCYAATTTACCTACAACAGTACCA	fragment D2 antisense

	GAGTGYAAGTGGTCACCACCAGACATACGYAAAGCTTT AGCYAAAACACGGAAGTGAATACCGTGGTTACGTTGAC	
oligoCSB863	AAGGGGTTGAAGACAACTACGTTGAAAAAGACCGTTCN CGTGGTATTTACTTCACTCAAGAC	fragment D3 F
oligoCSB914	GTTGGAAGACAAtcccGGTCTCCGGAGCGTTACCCCAA GGGTGACCYAAAGTACCACCACCGAACTGYAAACATGC GTCATCACCGAAG	fragment D3 R
oligoCSB870	CAGTGTTGGAAGACAATTCGAATTTGATACTATTGACA AATTRTAAtttttatttttcatgatgtttatgTG	fragment E1 F
oligoCSB915	AAGGGGTTGAAGACAATAGTCTTTTACACCGGCTTTGA ARCCAGCACCTGCTTTAGTTTC	fragment E1 R
oligoCSB916	AAGGGGTTGAAGACAAACTACCGTTTAACATTGAGACC TAGGGCAGTGAGCGCAAC	fragment E2 F
oligoCSB917	AGTGTTGGAAGACAAGAGCTTGAGTACAAGCTTCYAAA GCTACACGGTTAGCTGCAGCACCTGGAGGGAGACCCTA TGCGGCATCAGAG	fragment E2 R
oligoCSB918	GCTCGTAACGAAGGTCGTGACTTRGCTCGTGAAGGTGG CGACGTAATTCGTTCAGCTTGTAAATGGTCTCCAGAAT TRGCTGCTGCATGTGAAGTTTGGAAAGAAATTAAA	fragment E3 sense
oligoCSB919	CGAATTTAATTTCTTTCCAAACTTCACATGCAGCAGCY AATTCTGGAGACCATTTACAAGCTGAACGAATTACGTC GCCACCTTCACGAGCYAAGTCACGACCTTCGTTAC	fragment E3 antisense
**Amplification of *rbcL in* CSB426 transformants and Sanger sequencing**		
oligoCSB433	CCCTGACAGGAATATACATGGTT	*rbcL* locus F
oligoCSB933	GACAGGCAAATTTAAACAAAAGA	*rbcL* locus R

Table S4. Efficiency of chloroplast transformations

Construct	Unique codons	Modified codons	Modified bases	Transformants tested	Correct integration	Homoplasmy
**Recoding of *rpoA***						
pCSB245 (WT-FLAG)	57	-	-	22	19 (86%)	17 (89%)
pCSB246 (Def. comp.)	47	56 (7.5%)	99 (4.4%)	16	14 (87%)	14 (100%)
pCSB247 (ChimeraMAP)	48	176 (23.5%)	346 (15.4%)	16	10 (62%)	0 (0%)
pCSB248 (CUO)	44	176 (23.5%)	339 (15.1%)	16	12 (75%)	7 (58%)
pCSB322 (Def. comp.)	-	-	-	16	15 (95%)	15 (100%)
**Recoding of *ycf1***						
pCSB317 (WT)	59	-	-	6	6 (100%)	6 (100%)
pCSB416 (Def. comp.)	50	147 (7.1%)	245 (4.1%)	18	18 (100%)	14 (78%)
**Deletion of *psbA***						
pCSB323	-	-	-	22	19 (86%)	4 (21%)
**Restoration of *psbA***						
pCSB487 (WT)	37	-	-	4	4 (100%)	4 (100%)
pCSB488 (No introns)	37	-	-	6	6 (100%)	5 (80%)
pCSB489 (Def. comp., no introns)	34	16 (4.5%)	28 (2.6%)	3	3 (100%)	3 (100%)
**Recoding of *psaA***						
pCSB491	41	46 (6.1%)	91 (4%)	24	20 (83%)	20 (100%)
**Recoding of *trnM^e-psbE-rps9-ycf4-ycf3-rps18-rps2* operon**						
pCSB347	49	104 (6.1%)	174 (3.4%)	40	32 (80%)	32 (100%)
**Deletion of *rbcL***						
pCSB419	-	-	-	16	16 (100%)	16 (100%)
**Restoration of *rbcL***						
pCSB424	41	-	-	8	8 (100%)	8 (100%)
pCSB426	Combinatorial library			83	73 (88%)	73 (100%)
pCSB430	35	27 (5.7%)	48 (3.4%)	8	7 (87%)	7 (100%)

Table S5. ONT sequencing of recoded strains. Raw reads and genome *de novo* assemblies have been deposited in the European Nucleotide Archive, Project: PRJEB96787 (https://www.ebi.ac.uk/ena/browser/view/PRJEB96787). The data has also been deposited in Zenodo (DOI 10.5281/zenodo.17350679).

Strain	Run	Extraction method	SRE	Number of reads	Bases (Mb)	N50 (kb)	Chloroplast coverage	ENA accession	*De novo* assembly
CSB347_1	1	(9)	-	74.6 k	175.49	5.1	484x	ERR15743408	CDHCRX020000000
	2		+	29.5 k	129.68	9.8			
	3		+	60.3 k	199.23	6.9			
CSB416_1	1	(10)	-	18.2 k	121.61	23.9	53x	ERR15743409	OZ318231
CSB489_1	1	(9)	-	48.7 k	84.17	5.4	115x	ERR15743411	OZ318174
	2		-	70.6 k	123.03	5.3			
CSB491_2	1	(10)	-	7.6 k	39.44	19.8	92x	ERR15743412	CDGTVB020000000
	2		-	7.5 k	31.74	14.6			
	3		-	19.7 k	100.24	19.0			
	4		-	16.6 k	81.04	15.8			
CSB430_22	1	(10)	-	49.7 k	167.39	9.7	73x	ERR15743410	CDGTTB020000000

SI References

1. S. D. Gallaher, et al., High-throughput sequencing of the chloroplast and mitochondrion of *Chlamydomonas reinhardtii* to generate improved de novo assemblies, analyze expression patterns and transcript speciation, and evaluate diversity among laboratory strains and wild isolates. *Plant J.* 93, 545–565 (2018).
2. P. P. Chan, T. M. Lowe, tRNAscan-SE: Searching for tRNA genes in genomic Sequences. *Methods Mol. Biol.* 1962, 1–14 (2019).
3. V. Cognat, et al., PlantRNA, a database for tRNAs of photosynthetic eukaryotes. *Nucleic Acids Res.* 41, D273–9 (2013).
4. S. Alkatib, et al., The contributions of wobbling and superwobbling to the reading of the genetic code. *PLoS Genet*. 8, e1003076 (2012).
5. M. Fages-Lartaud, K. Hundvin, M. F. Hohmann-Marriott, Mechanisms governing codon usage bias and the implications for protein expression in the chloroplast of *Chlamydomonas reinhardtii*. *Plant J.* (2022). https://doi.org/10.1111/tpj.15970.
6. M. G. Kluesner, et al., EditR: A method to quantify base editing from Sanger sequencing. *CRISPR J.* 1, 239–250 (2018).
7. R. Sager, Inheritance in the green alga *Chlamydomonas reinhardi*. *Genetics* 40, 476–489 (1955).
8. H. O. Jackson, et al., CpPosNeg: A positive-negative selection strategy allowing multiple cycles of marker-free engineering of the *Chlamydomonas* plastome. *Biotechnol. J.* e2200088 (2022).
9. F. Chaux, N. Agier, S. Eberhard, Z. Xu, Extraction and selection of high-molecular-weight DNA for long-read sequencing from Chlamydomonas reinhardtii. PLoS One 19, e0297014 (2024).
10. R. J. Craig, A. R. Hasan, R. W. Ness, P. D. Keightley, Comparative genomics of Chlamydomonas. Plant Cell 33, 1016–1041 (2021).
